# Endogenous and exogeneous stimuli-triggered reactive oxygen species evoke long-lived carbon monoxide to fight against lung cancer

**DOI:** 10.1186/s12951-024-02688-x

**Published:** 2024-07-16

**Authors:** Yujia Fang, Jianjun Yang, Xiayi Liang, Jing Wu, Mengqing Xie, Kun Zhang, Chunxia Su

**Affiliations:** 1 Department of Medical Oncology, Shanghai Pulmonary Hospital, Tongji University Medical School Cancer Institute, School of Medicine, Tongji University, Shanghai, 200092 China; 2 Central Laboratory and Department of Orthopaedics, Shanghai Tenth People’s Hospital, School of Medicine, Tongji University, No. 301 Yan-Chang-Zhong Road, Shanghai, 200072 China; 3 Central Laboratory, Sichuan Academy of Medical Sciences, Sichuan Provincial People’s Hospital, University of Electronic Science and Technology of China, No. 32, West Second Section, First Ring Road, Chengdu, 610072 Sichuan China

**Keywords:** Reactive oxygen species, Long half-life, Lung cancer, Carbon monoxide, Cascade catalysis

## Abstract

Reactive oxygen species (ROS)-associated anticancer approaches usually suffer from two limitations, i.e., insufficient ROS level and short ROS half-life. Nevertheless, no report has synchronously addressed both concerns yet. Herein, a multichannel actions-enabled nanotherapeutic platform using hollow manganese dioxide (H-MnO_2_) carriers to load chlorin e6 (Ce6) sonosensitizer and CO donor (e.g., Mn_2_(CO)_10_) has been constructed to maximumly elevate ROS level and trigger cascade catalysis to produce CO. Therein, intratumoral H_2_O_2_ and ultrasound as endogenous and exogeneous triggers stimulate H-MnO_2_ and Ce6 to produce •OH and ^1^O_2_, respectively. The further cascade reaction between ROS and Mn_2_(CO)_10_ proceeds to release CO, converting short-lived ROS into long-lived CO. Contributed by them, such a maximumly-elevated ROS accumulation and long-lived CO release successfully suppresses the progression, recurrence and metastasis of lung cancer with a prolonged survival rate. More significantly, proteomic and genomic investigations uncover that the CO-induced activation of AKT signaling pathway, NRF-2 phosphorylation and HMOX-1 overexpression induce mitochondrial dysfunction to boost anti-tumor consequences. Thus, this cascade catalysis strategy can behave as a general means to enrich ROS and trigger CO release against refractory cancers.

**Graphic Abstract:**

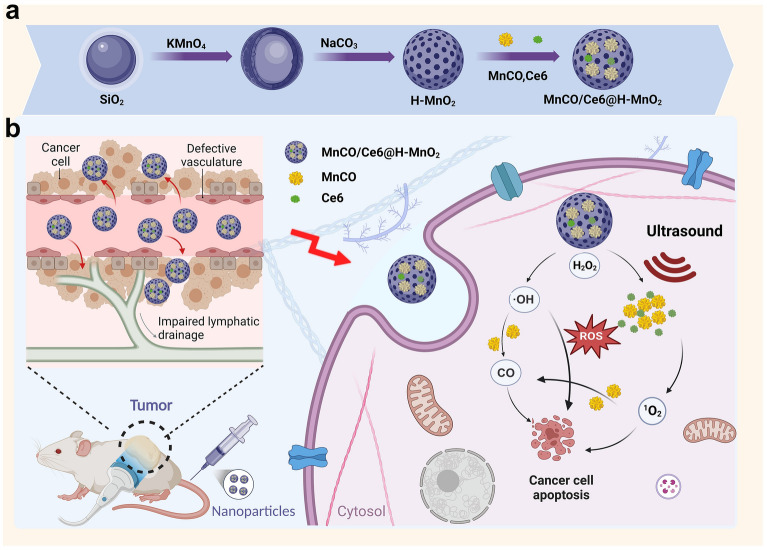

**Supplementary Information:**

The online version contains supplementary material available at 10.1186/s12951-024-02688-x.

## Introduction

Lung cancer is famous for high incidence and low cure rate, and has become the important cause of cancer-arsed deaths worldwide [1]. For intermediate and advanced cancers, the advent of targeted therapies and immunotherapies has improved the prognosis for most patients, but they still suffer from drug resistance and over-progression. The tremendous advances in nanomedicine and nanobiotechnology provide a highly promising route to address them [2, 3]. Especially, nanomedicine-assisted reactive oxygen species (ROS) anticancer treatments represented by chemodynamic therapy (CDT) [4], sonodynamic therapy (SDT) [5–8], photodynamic therapy (PDT) [9, 10], radiotherapy [11, 12], etc., have aroused increasing interests since they feature independence on tumor type and thus can serve as a general method. More significantly, rational nanobiotechnology design could re-programme tumor microenvironment to remove the resistance to the cancer therapy and magnify ROS-based therapy [10, 13–16]. Two major concerns in ROS-based anti-tumor therapy are the insufficient level of ROS and its short half-life, which ultimately determine the success or failure of such therapy. In most cases, efforts have been focused on enhancing the efficiency of ROS production, such as providing O2 to alleviate hypoxia [17] and using exogenous cavitation nuclei to enhance sonoluminescence [18, 19], combined dynamics therapy, etc. [20–23]. Nevertheless, the 2nd concern is usually overlooked. Although our team has previously reported a study in which ROS was converted into RNS with a half-life over 10^4^ times longer [24], the half-life of RNS still remains at around 10^–2^ s, which is insufficient for effectively interacting with oxidizing, and damaging DNA in cell nuclei and mitochondria before they are degraded.Moreover, that report failed to investigate how maximumly boost the ROS level.

As one of gas therapy, carbon monoxide (CO) treatment has been recorded to directly bring about cancer cell apoptosis by preventing mitochondria respiration and promoting the secretion of pro-apoptotic protein caspase3. More significantly, CO is physicochemically stable and can’t be annihilated, which means that it has a much longer half-life than ROS and RNS. Therefore, chasing for the transformation of short-lived and unstable ROS to long-lived and stable CO is an ideal option. In light of the fact that CO can bind to hemoglobin in red blood cells to result in normal tissue damages, precise and on-demand CO production at tumor is much preferable. It has documented that exogenous light trigger enabled CO release [25], which, nevertheless, is inapplicable for deep tumor due to the inherently poor penetration of light. Although the intratumoral H_2_O_2_ that could oxidize Mn_2_(CO)_10_ decomposition to release CO at tumor and enable endogenous stimuli-responsive precise CO release [26, 27], this process is regulated by H_2_O_2_ level and not all tumor sustains a high H_2_O_2_ level. More significantly, the oxidization activity of H_2_O_2_ is inferior to ROS, making CO release in a continuous manner, while this continuous release dictates that the accumulative CO concentration will not be high since CO can freely diffuse.

**Fig. 1:**
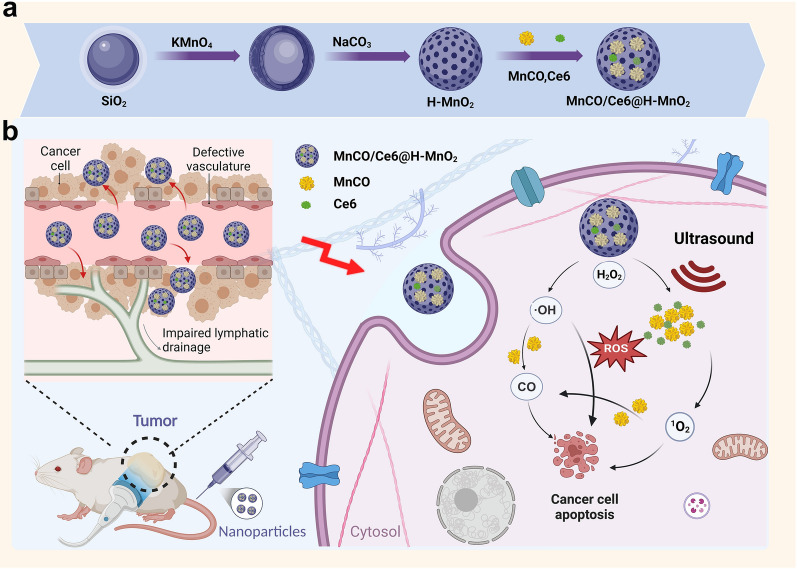
**Schematic procedures and the action rationales of such a endogenous and exogeneous stimuli-triggered cascade catalysis strategy for maximumly producing ROS and long-lived CO against lung cancer.** **a**.Schematic illustrations of the synthetic process of MnCO/Ce6@H-MnO_2_. **b**. Schematic procedures and the underlying rationales for an endogenous and exogenous stimuli-triggered cascade catalysis strategy.In light of the fact that the poor oxidation ability of intratumoral H_2_O_2_ failed to rapidly decompose MnCO precursors to release CO and enrich CO in tumor for executing anti-tumor actions, we designed cascade catalysis reactions consisting of endogenous H_2_O_2_-triggered CDT (producing •OH) and endogenous ultrasound-triggered SDT (producing ^1^O_2_) for initial ROS release and subsequent ROS oxidation-induced MnCO decomposition for CO production. Additionally, in light of the short life of ROS (e.g., ^1^O_2_: 10^–6^ s, •OH: 10^–9^ s), the partial conversion of ROS into long-term stable CO can significantly prolong the life to prohibit the annihilation before they could reach mitochondria or cell nuclei to oxidize and destroy DNA, which have rarely received attentions. More significantly, CO therapy circumvented the inherent resistance singling axis of tumor against in ROS therapy since no resistance singling pathway associated with CO therapy has not been found yet.

To cope with these issues, we designed and engineered an endogenous and exogeneous stimuli-responsive sequential therapeutic nanoplatform based on mesoporous hollow manganese dioxide (H-MnO_2_), where H-MnO_2_ served as the carrier to load sonosensitizer Chlorin e6 (Ce6) and CO donor (e.g., manganese carbonyl) (Fig. 1a). Under exogenous ultrasound and endogenous H_2_O_2_ triggers, singlet oxygen (^1^O_2_) and hydroxyl radicals (•OH) via Ce6-mediated SDT and H-MnO_2_-mediated CDT, respectively, were accessible in response to acidic tumor microenvironment-arised H-MnO_2_ decomposition, which could significantly elevate the accumulative ROS level, addressing the 1st concern (i.e., low ROS level) in the ROS-based anti-tumor therapy and breaking the penetration depth limitation for PDT (Fig. 1b). More significantly, the oxidation reaction between ROS and Mn_2_(CO)_10_ was anticipated, and the conversion of partial ROS into CO was accessible since ROS featured a much higher oxidation activity than H_2_O_2_, addressing the 2nd concern (i.e., short ROS half-life) (Fig. 1b). The cascade catalytic reaction between Mn_2_(CO)_10_ and ROS initiated the liberation of CO to effectively convert short-lived ROS into long-term stable CO, which not only left enough time to diffuse into cell nuclei and mitochondria to oxidize and destroy DNA, but also circumvented the inherent resistance singling axis associated with ROS therapy and heightened tumor susceptibility. Consequently, these action rationales synergistically boosted the efficacy of SDT/CDT/CO therapy and significantly delayed lung cancer regression, recurrence and metastasis with the prolonged survival rate.

It has recorded that CO could inhibit the key regulator of redox homeostasis maintenance (i.e., cystathionine beta synthase (CBS)) and attenuate the tolerance to ROS therapy [28]. Given it, the long-lived CO along with residual ^1^O_2_ and •OH was expected to fight against lung cancer with the elevated safety profile via activating Akt signaling pathway including AKT-1, HMOX-1 and NRF-2 and facilitating intracellular communication, and prolong the survival rate associated with tumor recurrence and metastasis inhibition (Fig. 1b). The groundbreaking research represents a novel application of nanomedicine in the treatment of lung cancer, offering valuable insights for clinical management and potential applicability to other types of cancer.

## Materials and methods

### Reagents

**Table:** 

Reagents	Suppliers
Treaethyl orthosilicate (TEOS)	Aladdin Reagent Co. LTD (Shanghai, China)
Potassium permanganate (KMnO_4_)	Sinopharm Chemical Reagent CO., Ltd. (China)
4,6-diamino-2-phenylindole (DAPI)	Sigma-Aldrich Trading Co., Ltd. (Shanghai, China)
Ki-67 assay kit	Sigma-Aldrich Trading Co., Ltd. (Shanghai, China)
Hydrogen peroxide solution (H_2_O_2_, 30 wt%)	J&K chemical CO. LTD
Triton X- 100	Sigma-Aldrich Trading Co., Ltd. (Shanghai, China)
Cell Counting Kit-8 (CCK-8) assay kit	Dojindo Laboratories (Kumamoto, Japan)
CO probes	Xi'an Ruixi Biotechnology Co., Ltd
Enzyme-linked immunosorbent assay (ELISA) kits	Cell Signaling Technology, Inc (MA, USA)
Fluorescein isothiocyanate (FITC)	Sigma-Aldrich Trading Co., Ltd. (Shanghai, China)
Dulbecco’s modified Eagle medium (DMEM)	Thermo Scientific (Beijing, China)
Glutathione (GSH)	Aladdin Reagent Co. LTD (Shanghai, China)
Sodium carbonate (Na_2_CO_3_)	Sinopharm Chemical Reagent CO., Ltd. (China)
Manganese carbonyl (termed as MnCO)	Sigma-Aldrich Trading Co., Ltd. (Shanghai, China)
amine-cyanine 5.5 (Cy5.5)	Sigma-Aldrich Trading Co., Ltd. (Shanghai, China)
Penicillin and streptomycin	Thermo Scientific (Beijing, China)
Chlorin e6 (Ce6)	J&K chemical CO. LTD
propidium iodide (PI) and calcein acetoxymethyl ester (Calcein-AM)	Sigma-Aldrich Trading Co., Ltd. (Shanghai, China)
Terminal deoxynucleotidyl transferase-mediated dUTP-biotin nick end labeling (TUNEL) kit	Sigma-Aldrich Trading Co., Ltd. (Shanghai, China)
BCA protein assay kit	Dojindo Laboratories (Kumamoto, Japan)
Fetal bovine serum (FBS)	Thermo Scientific (Beijing, China)
2′,7′-dichlorofluorescin diacetate (DCFH-DA)	Sigma-Aldrich Trading Co., Ltd. (Shanghai, China)
Deionized (DI) water (18.2 MΩ cm)	Our laboratory
dihydroethidium (DHE)	Sigma-Aldrich Trading Co., Ltd. (Shanghai, China)
Hydrogen Peroxide Testing Kit	Beijing Solarbio Science & Technology Co.,Ltd

### Synthesis of MnCO/Ce6@H-MnO_2_

Pure silica nanospheres (sSiO_2_) could be fabricated through a classic method [29]. Subsequently, KMnO_4_ (300 mg) solution was dropped into the sSiO_2_ dispersion (40 mg) under ultrasound oscillation, and reacted for 6 h. Afterwards, the precipitates were collected through centrifugation at 14,800 revolutions per minute. The obtained MnO_2_-incorprated sSiO_2_ was re-dispersed in sodium carbonate solution (2 mol/L) at 60 °C for a half day, followed by centrifugation and rinsing with water several times to obtain H-MnO_2_. As for Ce6 (0.2 mg/mL) and Mn_2_(CO)_10_ (0.6 mg/mL) co-entrapment, H-MnO_2_ dispersions (0.2 mg/mL) were incubated with Ce6 and MnCO for 24 h, obtaining MnCO/Ce6@H-MnO_2_.

### Mn_2_(CO)_10_ loading capacity

The MnCO loading capacity of H-MnO_2_ NPs was measured using a scanning spectrometer (DU730, Beckman, USA). In short, the supernatant harvested after centrifugation and rinsing with MeOH during above Mn_2_(CO)_10_ loading was gathered, and then screened on the scanning spectrometer where the absorbance intensity at 340 nm was obtained. The Mn_2_(CO)_10_ entrapment ratio was obtained through the subsequent formula: Entrapment ratio(%) = (N–N1)/N2*100%, where N indicates the initially-added mass of Mn_2_(CO)_10_, N1 indicates the Mn_2_(CO)_10_ content in the supernatant, and N2 indicates the obtained H-MnO_2_ content.

### In vitro CO burst survey

CO burst from Mn_2_(CO)_10_ or MnCO/Ce6@H-MnO_2_ NPs was measured according to the previous reported [25, 30]. Firstly, bovine hemoglobins were added to deoxygenated PBS, followed by complete dissolution, and then sodium dithionite was added into the solution and reduced hemoglobin. Afterwards, 200 μL of Mn_2_(CO)_10_ or MnCO/Ce6@H-MnO_2_ NPs solutions were blended with aforementioned solution. The UV–vis absorption spectra of MnCO/Ce6@H-MnO_2_ NPs were recorded every 5 min using a scanning spectrometer (DU730, Beckman, USA). The levels of CO burst were obtained according to the following formula: L_co_ = L_Hb_ (528.6*I_410nm_-304*I_430nm_)/(216.5*I_410nm_ + 442.4*I_430nm_), wherein L_CO_ is the level of CO burst, L_Hb_ is the level of bovine hemoglobins, and I_410nm_ and I_430nm_ represent the absorbance of the blended solution at the wavelength of 410 nm and 430 nm, respectively.

### Cell lines

Three cell lines, i.e., mouse Mouse lung epithelial cells (TC-1), fibroblasts cell line (L929 cells) and the mouse lung adenocarcinoma cell line (Lewis cells) were obtained from the Cell Bank of Shanghai Institutes for Biological Sciences, Chinese Academy of Sciences. All cells were cultured in DMEM containing 10% fetal bovine serum (FBS) and 0.1% penicillin–streptomycin at 37 ºC with 5% CO_2_.

### In vivo cancer model establishment

BALB/c mice aged at 4–5 weeks were purchased from Shanghai Slac Laboratory Animal Co., Ltd.. All animal experiments were conducted under the protocols approved by the Institutional Animal Care and Use Committee (IACUC) of Medicine of Tongji University Affiliated Shanghai Pulmonary Hospital (Animal Welfare Ethics acceptance number No: k20-197Y). To establish the lung cancer model, 200 μL of Lewis cells (1.5 × 10^7^) were administered into the right armpit of above mice. As the tumor volume approached 60–80 mm^3^, in vivo animal experiments were enforced. If the tumor burden exceeded 1500 mm^3^, the mice were euthanized based on animal ethics demands.

### In vivo synergistic therapeutic effect

Lewis tumor-bearing mice (~ 100 mm^3^) were divided into six groups (5 mice for each group) and the treatment was set as follows: Group 1: PBS; Group 2: H-MnO_2_ (0.5 mg/kg); Group 3: Ce6@H-MnO_2_ (Ce6: 3 mg/kg); Group 4: Ce6@H-MnO_2_ + US (Ce6: 1 mg/kg, US: 1.0 MHz, 1.0 W/cm^2^, 60 s); Group 5: MnCO/Ce6@H-MnO_2_ (Ce6: 3 mg/kg, Mn: 5 mg/kg); Group 6: MnCO/Ce6@H-MnO_2_ + US (Ce6: 1 mg/kg, Mn: 5 mg/kg, US: 1.0 MHz, 1.0 W/cm^2^, 120 s). Different samples were intravenously administered into the corresponding mice in above set groups. These tumor-bearing mice underwent thrice independent treatments on days 0, 2, and 4. All mice received ultrasound treatment on days 1, 3, and 5. Afterwards, the width (W) and length (L) of tumor in each mouse was measured once per two days to obtain the tumor volumes according to the formula: (L × W^2^)/2, with which the body weight of each mouse was weighed. When time come to the termination of experiments, all mice were killed to isolate their tumor and carry on weighing. Some main organs including heart, spleen, lung, liver, and kidney were collected for hematoxylin and eosin (H&E) staining, TUNEL staining as well as Ki-67 staining, respectively. Simultaneously, blood was acquired from treated mice in different groups for various inspections.

### DHE staining procedures for frozen sections

1. Staining: Thaw the frozen sections, delineate the tissue with a histochemical pen, and incubate at 37 ℃ in a light-protected environment for 30 min using PBS-diluted DHE.
2. Nuclear staining: Transfer the slides to PBS (pH 7.4) on a decolorizing shaker and gently agitate in darkness for three washes of 5 min each. After briefly air-drying the sections, apply DAPI staining solution at room temperature and incubate in darkness for 10 min to label the nuclei.
3. Mounting: Rinse the slides on a decolorizing shaker with PBS (pH 7.4) for three washes of 5 min each while avoiding exposure to light. After partially drying the sections, mount them using an anti-fade mounting medium suitable for fluorescence microscopy.
4. Microscopic examination and imaging: Examine and capture images of the sections under an inverted Nikon fluorescence microscope.

### Statistical analysis

Data are expressed as Mean ± standard deviation (SD, n = 3–8). The statistical significances between two groups were implemented using the classic Student’s t-test, and *P < 0.05, **P < 0.01, and ***P < 0.001.

## Results

### Design and construction of MnCO/Ce6@H-MnO_2_

**Fig. 2:**
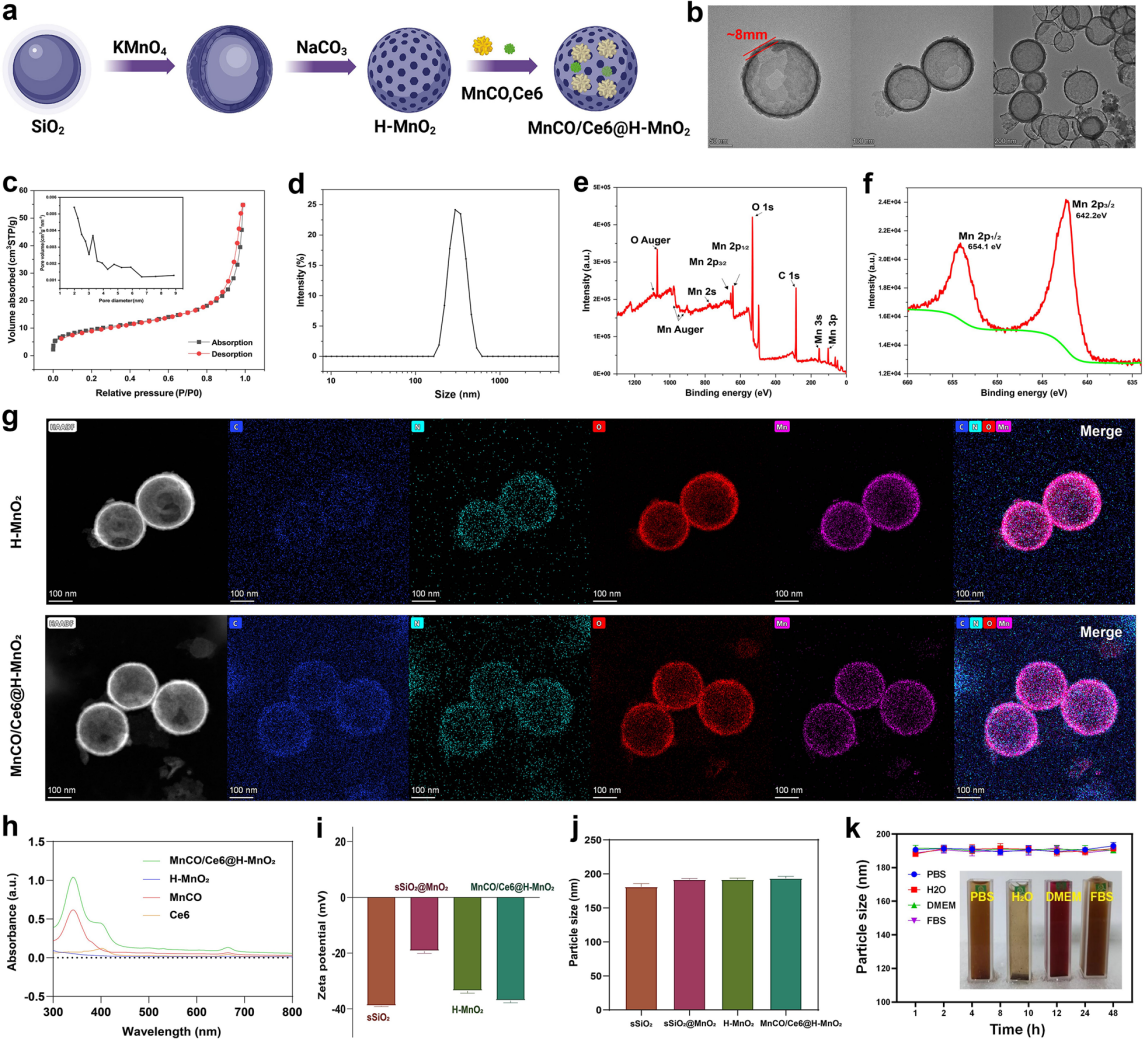
Characterizations of H-MnO_2_ and MnCO/Ce6@H-MnO_2_. **a** Schematic for understanding the construction steps of H-MnO_2_ and MnCO/Ce6@H-MnO_2_. **b** TEM images of H-MnO_2_ NPs. **c** N_2_ adsorption/desorption isotherms and pore diameter distribution curve (inset) of the H-MnO_2_ carriers. **d** Size distribution curve of H-MnO_2_ nanoparticles in water. **e**, **f** Wide-band (**e**) and narrow-band Mn2p **f** XPS spectra of H-MnO_2_ nanoparticles. **g** High-angle annular dark field (HAADF)- scanning transmission electron microscope (STEM) image and elemental mapping images of H-MnO_2_ and MnCO/Ce6@H-MnO_2_. **h** UV–vis absorption spectra of Ce6, MnCO, H-MnO_2_ and MnCO/Ce6@H-MnO_2_. **i**, **j** The zeta potential and particle sizes of MnCO/Ce6@H-MnO_2_ and its intermediates. **k** Time-dependent particle size variation profiles of MnCO/Ce6@H-MnO_2_ nanoparticles in several varied media including PBS, H_2_O, DMEM and FBS. Date are presented as means ± standard deviation (s.d.) (n = 3)

The synthetic process and details of MnCO/Ce6@H-MnO_2_ nanoparticles are shown in Figs. 1a and 2a, wherein H-MnO_2_ were firstly obtained according to the classic templating etching method [29], followed by the co-loading of Ce6 and Mn_2_(CO)_10_. Monodispersed silica nanoparticles (MSNs) as templates show an average particle size at 180 nm (Figure S1), which determines the size of ultimate H-MnO_2_. After reaction with potassium permanganate (KMnO_4_) solution, MnO_2_ components are intercalated to obtain SiO_2_@MnO_2_ with a diameter of 200 nm. Subsequently, the H-MnO_2_ nanospheres are obtained by removing SiO_2_ components, and the as-prepared H-MnO_2_ have a regular spherical morphology whose shell thickness is ~ 8 nm (Fig. 2b). s-SiO_2_ removal by etching leaves a large number of mesopores with a pore size of 3.2 nm and concurrently imparts H-MnO_2_ with large surface area (Fig. 2c), providing adequate space for accommodating Ce6 and Mn_2_(CO)_10_. The H-MnO_2_ carriers have the most probable size at 200 nm ~ 300 nm (Fig. 2d). Energy diffraction spectrum (EDS) indicates the atomic ratio of Mn/O at 1:2, denoting the + 4 valence of Mn (Figure S2), and X-ray photoelectron spectroscopy (XPS) test further verifies the valence of Mn atoms (Fig. 2e,f) [4]. The surface Zeta potential varies from s-SiO_2_ to s-SiO_2_@MnO_2_, and further to H-MnO_2_, indicating the successful synthesis of H-MnO_2_ carriers.

After co-loading Ce6 and Mn_2_(CO)_10_, no evident structural and compositional alterations are found, as evidenced by the HAADF and mapping images of compositional elements (Fig. 2g). The characteristic peaks of Ce6 and Mn_2_(CO)_10_ at 400 nm and 330 nm in MnCO/Ce6@H-MnO_2_ unveiling the successful co-entrapment of Mn_2_(CO)_10_ and Ce6 **(**Fig. 2h**)**, respectively. As well, the zeta potential declines after co-loading Mn_2_(CO)_10_ and Ce6 (Fig. 2i), while the co-loading fails to alter the colloidal particle size (Fig. 2j), which means that Mn_2_(CO)_10_ and Ce6 predominantly reside in hollow cavity and mesopores in shell of H-MnO_2_ carriers. Significantly, the loading capacities of Ce6 and Mn_2_(CO)_10_ can be adjusted based on their feeding ratios to H-MnO_2_ carriers (Figures S3 and S4), wherein the largest Mn_2_(CO)_10_ and Ce6 percentages correspond to 40.5% and 32.6%, respectively. In addition, to determine the drug adsorption time, we observed the drug loading capacity of the nanomaterials after various soaking durations. The results demonstrated that saturation was achieved within approximately 24 h. Consequently, this specific drug loading condition was selected for subsequent experiments (Figure S5).

Intriguingly, such nanoplatforms exhibit high colloidal stability in different media due to no evident alterations in the hydrodynamic size (Fig. 2k). To further demonstrate the material’s stability, we have included numerical images before mixing, after mixing, and after 24 h of mixing (Figure S6).These results indicate that the H-MnO_2_ featuring porous outer layer is an appropriate vehicle for transporting anticancer therapeutic agents.

### Endogenous and exogenous stimuli-triggered ROS production and CO release

**Fig. 3:**
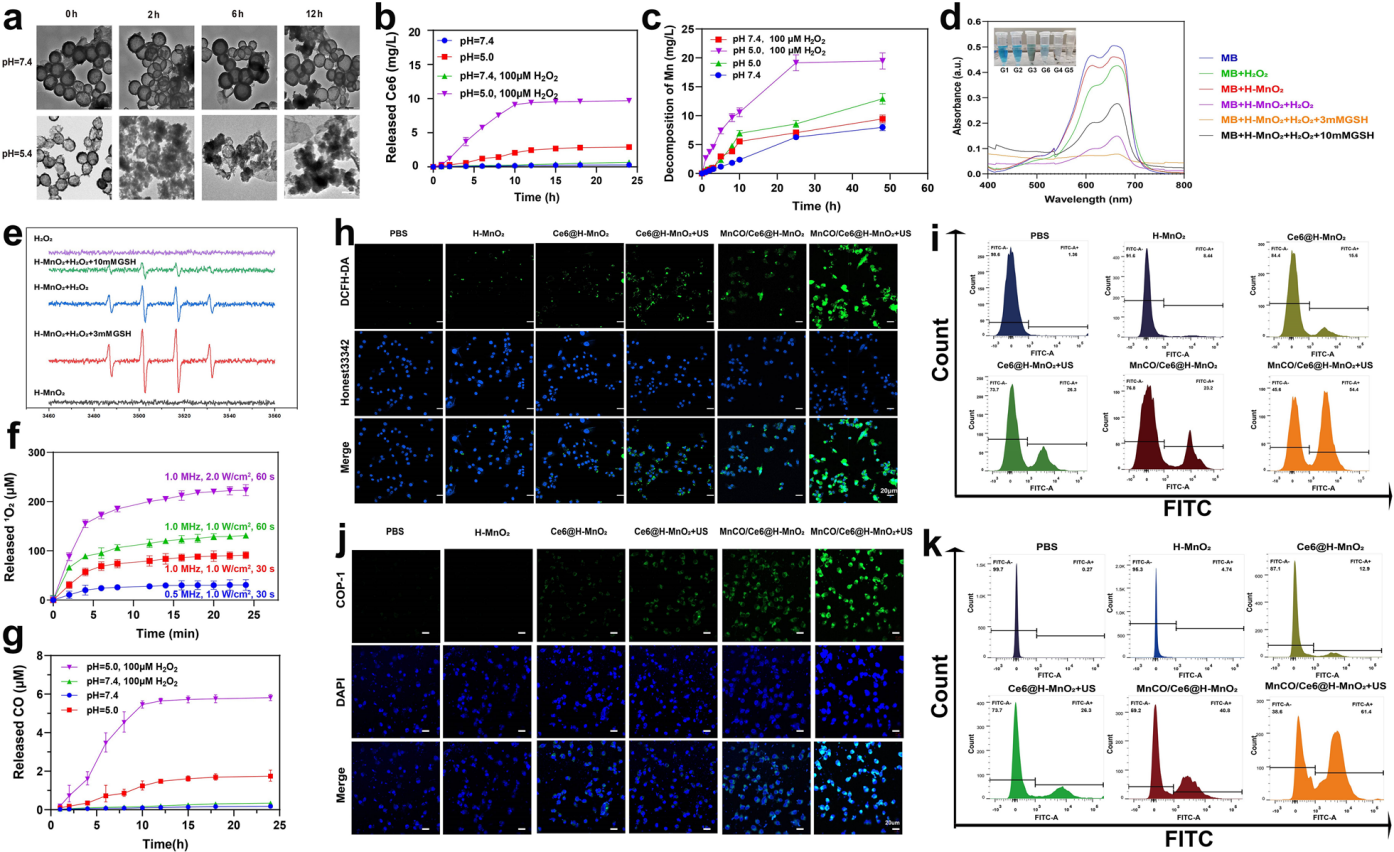
Sequential CDT and SDT-mediated ROS production and CO release from MnCO/Ce6@H-MnO_2_ in vitro. **a** TEM images of H-MnO_2_ after incubation in buffer with different pH values (7.4 and 5.4) as a function of time. **b** In vitro accumulative release percentage of Ce6 from MnCO/Ce6@H-MnO_2_ at different pH values with varied doses of H_2_O_2_. **c** The release profile of Mn from MnCO/Ce6@H-MnO_2_ (5 mg/mL). **d** UV–Vis absorption spectra of MB that underwent different treatments. **e** ESR spectra of •OH in different reaction systems. **f** The release of ^1^O_2_ at different Ultrasonic condition. **g** In vitro accumulative burst amount of CO from MnCO/Ce6@H-MnO_2_ at different pH values with varied doses of H_2_O_2_. **h** Confocal laser scanning microscopic (CLSM) images of Lewis cells after various treatments for monitoring ROS using DCFH-DA as the ROS probe and Honest33342 as nuclear staining dye. **i** FCM patterns of Lewis cells that experienced staining with DCFH-DA after above various treatments. **j** CLSM images of Lewis cells after various treatments for monitoring intracellular CO level with COP-1 as the CO fluorescence probe. **k** FCM patterns of Lewis cells that experienced staining with COP-1 after above various treatments. H-MnO_2_: 100 μg/mL; US parameters: 1.0 MHz, 1.0 W/cm^2^, 15 s per time with 4 times for 60 s in total. Date are presented as means ± s.d. (n = 3)

Manganese dioxide (MnO_2_)-based nanomaterials are known to be unstable and could be dissociated into Mn^2+^ at low pH, which further reacted with intratumoral H_2_O_2_ to give birth to •OH for implementing CDT [31–34]. To verify it, the structure of H-MnO_2_ after storage in PBS with varied pH values as a function of time was traced. After 12 h, no evident alterations in structure and morphology suggest the high structural stability of H-MnO_2_ at pH = 7.4 solution. In contrast, H-MnO_2_ exhibits a time-correlated degradation manner under acidic conditions (pH = 5.4) because of the dissociation of H-MnO_2_ into Mn^2+^, and more fragments are found as the time elapses (Fig. 3a). Quantitative release assays reveal the structural collapse of H-MnO_2_ in the presence of low pH values and rich H_2_O_2_ allows more releases of Mn^2+^ and Ce6 release since H_2_O_2_-abundant acidic tumor microenvironment favors more robust redox reaction (Fig. 3b, c).

H_2_O_2_ and GSH levels are higher in tumors than in normal tissue due to insufficient blood replenishment inside solid tumors [35, 36]. Inspired by it, Fenton-like reaction mediated by Mn^2+^ is within easy reach. To verify it, the ability of Mn^2+^ to induce CDT by converting H_2_O_2_ into •OH to degrade methylene blue (MB) was assessed. A drastic drop in the characteristic peak of MB is found upon reaction with H_2_O_2_ for 30 min, demonstrating •OH production and CDT occurrence that induces MB decolorization. Especially after adding 3 mM glutathione (GSH), more •OH production accounts for the complete color recession and the lowest absorbance of MB since GSH can promote H-MnO_2_ decomposition into Mn^2+^ to trigger Fenton-like reaction. However, when the GSH dose is increased to 10 mM, excessive GSH depletes the generated •OH, thereby inhibiting the oxidation of MB by •OH and resulting in the presence of unoxidized MB. (Fig. 3d). Similar results are obtained using electron spin resonance (ESR) technology, wherein the characteristic ESR peak of •OH is significantly enhanced in the group of H-MnO_2_ + H_2_O_2_, and reaches the highest level when adding 3 mM GSH, but decrease in the group of 10 mM GSH because of the excessive GSH-arised ROS exhaustion (Fig. 3e).Subsequently, the Ce6-mediated SDT process was evaluated, and the production of ^1^O_2_, a hallmark of SDT, was monitored using the singlet oxygen sensor green (SOSG). Higher ultrasound power density and longer treatment time were applied to enhance the generation of ^1^O_2_, demonstrating the occurrence of sonocatalytic process(Fig. 3f). Thanks to the large quality of ROS production, the reaction between ROS and Mn_2_(CO)_10_ is encouraged since ROS including ^1^O_2_ and OH is equipped with a more potent oxidation ability. Results also show that the acidic and H_2_O_2_-rich conditions support the most ROS production to stimulate more CO release from MnCO/Ce6@H-MnO_2_ (Fig. 3g), successfully switching the partial short-lived ROS to long-lived CO, which will boost the anti-tumor efficiency of ROS-based therapy. In order to mitigate the potential impact of ultrasound on carbon monoxide release, we have incorporated experimental figure S7 as additional evidence to substantiate that ultrasound does not induce Mn_2_(CO)_10_ liberation.

In the cellular-level experiments, further monitoring on ROS and CO levels were carried out. A ROS indicator, 2',7'-Dichlorodihydrofluorescein diacetate (DCFH-DA) was used [37], and the MnCO/Ce6@H-MnO_2_ + US harvest the strongest ROS signal, suggesting the most ROS accumulation via the CDT and SDT processes (Fig. 3h). Noticeably, Mn_2_(CO)_10_ loading provides more Mn source to trigger CDT, resulting more ROS, as evidenced by the comparison between Ce6@H-MnO_2_ and MnCO/Ce6@H-MnO_2_. Quantitative results also reflect the variation trend of ROS level via flow cytometry (FCM), wherein ROS-positive cells increase and the treatment with MnCO/Ce6@H-MnO_2_ + US reaches the highest ROS level (Fig. 3i). Afterwards, a classical carbon monoxide fluorescence probe (COP-1) was adopted to examine the intracellular ROS-catalyzed carbon monoxide production. The fluorescence intensity gradually increases as the ROS level rises, and the MnCO/Ce6@H-MnO_2_ + US group harvest the highest green fluorescence signal, represented the most CO contents, which can be attributed that the most ROS accumulation stimulated the most decomposition of Mn_2_(CO)_10_ into CO (Fig. 3j, S8, S9). Apart from it, FCM analysis was used to further quantitatively examine CO release, and identical results are obtained (Fig. 3k). All these results provide reliable and adequate evidences of resolving the two concerns that ROS-based anti-tumor therapy encounters: maximumly elevating ROS level via the SDT and CDT combined strategy and prolonging the half-life via switching ROS to CO.

### In vitro anti-tumor evaluation

Contributed by the considerably-elevated ROS accumulation and CO release, desirable anti-tumor outcomes can be expected. Prior to assessing it, the in vitro biocompatibility of H-MnO_2_ carriers was evaluated on normal L929, TC-1 and Lewis cells via the CCK-8 assay. A neglectable cytotoxicity is found on three cells under neutral and acidic conditions even though H-MnO_2_ dose reaches 300 μg/mL (Figure S10), denoting the excellent biosafety of H-MnO_2_ nanoplatforms. This phenomenon also suggests the presence of heterogeneity-derived inadequate H_2_O_2_ in Lewis cells, resulting in poor CDT process. This point has been validated in ROS assay (Fig. 3h, i) wherein H-MnO_2_-mediated CDT alone fails to generate sufficient ROS when comparing with Control group.

**Fig. 4:**
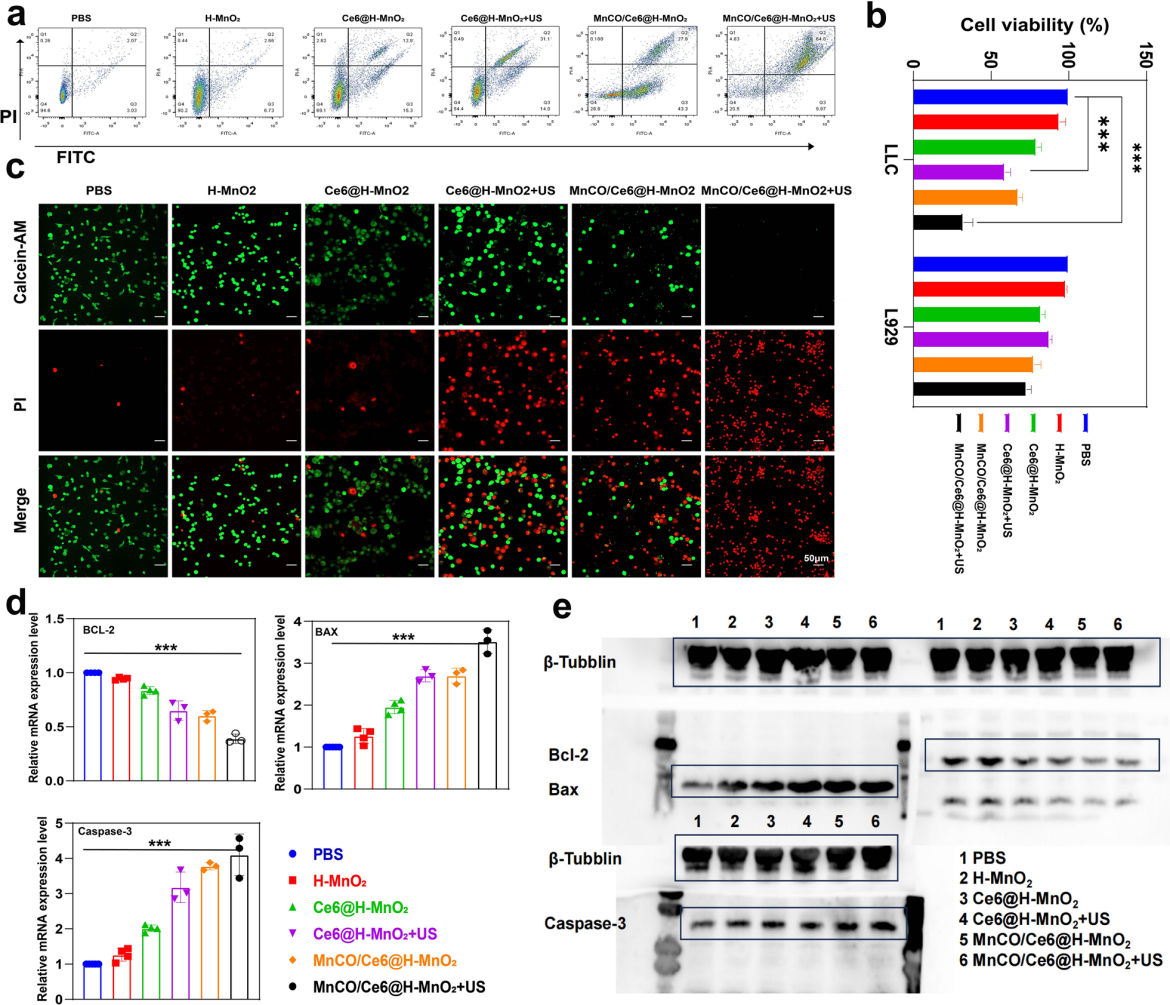
In vitro anti-tumor evaluations based on the maximumly-elevated ROS accumulation and long-lived CO release cascade catalysis effects. **a** FCM data for analyzing annexin V-FITC/PI-stained Lewis cells after undergoing various treatments and evaluating their apoptosis. **b** Relative viabilities of Lewis and L929 cells after various treatments. **c** CLSM images of calcein-AM and PI co-stained Lewis cells after different treatments. **d** Relative mRNA transcription levels of Bcl-2, Bax and Caspase-3 in Lewis cells that underwent varied treatments. **e** Western blot bands of Bcl-2, Bax and Caspase-3 expression in Lewis cells that underwent varied treatments. Note, H-MnO_2_: 100 μg/mL; US parameters: 1.0 MHz, 1.0 W/cm^2^, 15 s per time with 4 times for 60 s in total. Data presented as mean ± s.d. (n = 3). The statistical significance of differences was conducted with a Student’s t-test, and ***P < 0.001

Depending on the supplementary ROS induced by SDT process, the MnCO/Ce6@H-MnO_2_ + US group acquires the most ROS accumulation and evokes the highest CO release, thus successfully inducing the most deaths (over 70%) of Lewis cells via FCM analysis (Fig. 4a). Beyond that, CCK8 tests were adopted to further confirm this point. After different treatments, the constructed nanoplatforms exert the strongest killing effect on mouse tumor cells (Lewis cells), but have no influences on normal L929 cells (Fig. 4b). Similar results are further obtained via the calcein AM/PI co-staining tests. A large area of red fluorescence signal illuminates the Lewis cells in MnCO/Ce6@H-MnO_2_ and Ce6@H-MnO_2_ + US groups after 24 h of processing (Fig. 4c), representing the dead cells, which was further confirmed by quantitative analysis to cause tumor cell death(Figure S11). In particular, almost all Lewis tumor cells are dead upon treatment with MnCO/Ce6@H-MnO_2_ + US, but the treatment fails to kill L929 cells (Figure S12a), indicating the excellent safety and treatment precision.Meanwhile, we conducted verification of the mRNA transcription levels and protein expression levels of apoptosis-related genes through RT-qPCR and Western blot, aiming at provide further confirmation of the material's safety (Figure S12b-d).

### Mechanistic explorations

To understand the killing mechanism, real-time quantitative polymerase chain reaction (RT-qPCR) survey was firstly harnessed to trace mRNA transcription level of some pivotal apoptosis-associated genes. Bcl-2 mRNA expression is down-regulated in all treated groups, and the largest decline magnitude occurs to the MnCO/Ce6@H-MnO_2_ + US group (Fig. 4d). Meanwhile, MnCO/Ce6@H-MnO_2_ + US triggers the highest mRNA transcription level of pro-apoptotic genes including Bax and Caspase-3 (Fig. 4d). Western blot (WB) assays further clarifies the apoptotic signaling pathway (Fig. 4e). The treatment of MnCO/Ce6@H-MnO_2_ + US harvests the most upregulation of Bax and Caspase-3 proteins and coincidently significantly instigates Bcl-2 downregulation, and this result is in accordance with RT-qPCR analysis.

**Fig. 5:**
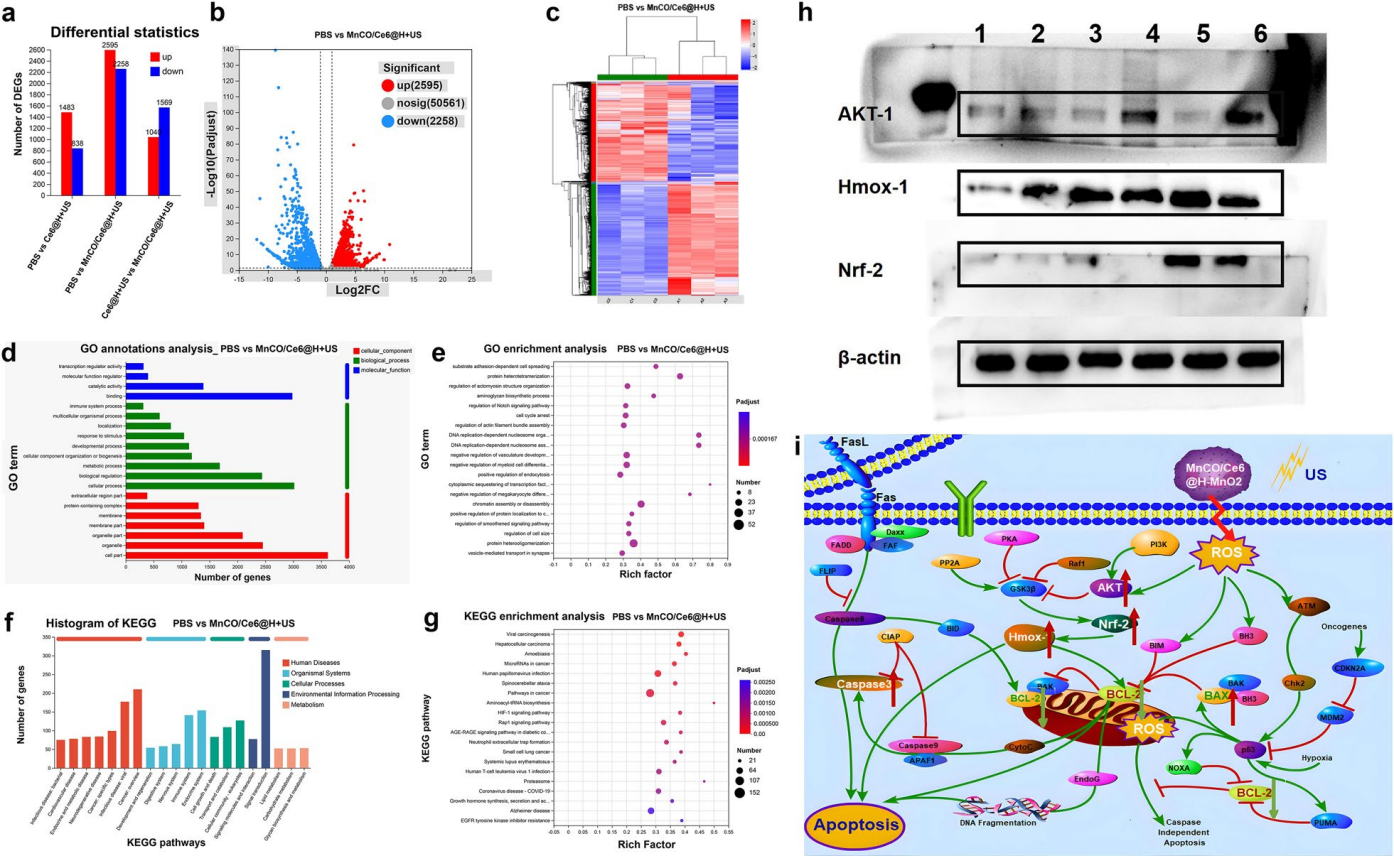
Mechanistic explorations of anti-tumor therapy via RNA sequencing and WB analysis. **a** Statistical plot of expression differences between 3 groups. **b** Volcano plot of expression differences between PBS group and MnCO/Ce6@H-MnO_2_ + US group. **c** Heatmap of differentially expressed genes. **d** GO classification statistics map of differential genes. **e** Results of GO enrichment analysis of differential genes. **f** KEGG classification statistics map of differential genes. **g** Results of KEGG enrichment analysis of differential genes. **h** WB bands of AKT-1, HMoX-1 and NRF-2 proteins in Lewis cells after experiencing varied treatments. **i** Signaling pathways of such a maximumly-elevated ROS accumulation and long-lived CO release cascade catalysis-unlocked anti-tumor therapy. H-MnO_2_: 100 μg/mL; US parameters: 1.0 MHz, 1.0 W/cm^2^, 15 s per time with 4 times for 60 s in total

In order to further uncover the signaling pathway, more systematic examinations were carried on, and RNA sequencing and bioinformatics analysis were implemented to explore the possible action mechanisms. A large difference in RNA level expression among the PBS, Ce6@H-MnO_2_ + US and MnCO/Ce6@H-MnO_2_ + US groups is found (Fig. 5a), and Fig. 5b, c clearly show the significantly differential gene profiles between the MnCO/Ce6@H-MnO_2_ + US and PBS groups. The classification and enrichment analysis of differential genes according to Gene Ontology (GO) / Kyoto Encyclopedia of Genes and Genomes (KEGG) show that the expression of mitochondrial function-related genes in experimental group tremendously varies in comparison to that in the control group (Fig. 5d–g). In detail, Akt signaling pathway is activated, and the down-stream targets including AKT-1, Nrf-2 and HMOX-1 are highly expressed after the activated genomic communication. This result reveals that the treatment system may activate the Akt signaling pathway to induce apoptosis. Furthermore, the molecular proteins of Akt signaling pathway, AKT-1, Nrf-2 and HMOX-1, proceeded to be inspected by WB, and they are found to be highly expressed in the MnCO/Ce6@H-MnO_2_ + US group, which is consistent with the RNA sequencing results (Fig. 5h).

Collectively, the detailed signaling pathway of such a maximumly-elevated ROS accumulation and long-lived CO release cascade catalysis-unlocked anti-tumor therapy is outlined after taking all above together, as shown in Figs. 1b and 5i. The synthesized MnCO/Ce6@H-MnO_2_ in an acidic environment can trigger a cascade of reactions that promotes the generation of •OH and ^1^O_2_ via enhanced CDT and SDT processes, thereby stimulating long-lived CO release via ROS-based redox reaction with Mn_2_(CO)_10_ and remodeling the genomic instability-induced tolerance to ROS therapy. By activating these damaged mitochondria, the released CO and ROS resulted in Akt signaling pathway activation represented by AKT-1, Nrf-2 and HMOX-1, thereby improving the therapeutic efficiency. Probably, there are other potential emerging pathways or cell subtypes that manipulate the CO/ROS anti-tumor process due to the tumor spatial–temporal heterogeneity, which, however, can’t be discerned due to the limitation of common RNA-Seq technology. Fortunately, the unwavering advancements in single-cell analysis technology have provided indispensable support for cutting-edge medical scientific exploration [38, 39], which will reveal more anti-tumor mechanisms of this endogenous and exogeneous stimuli-triggered cascade catalysis method.

### In vivo anti-tumor evaluation based on MnCO/Ce6@H-MnO_2_ nanoplatforms

**Fig. 6:**
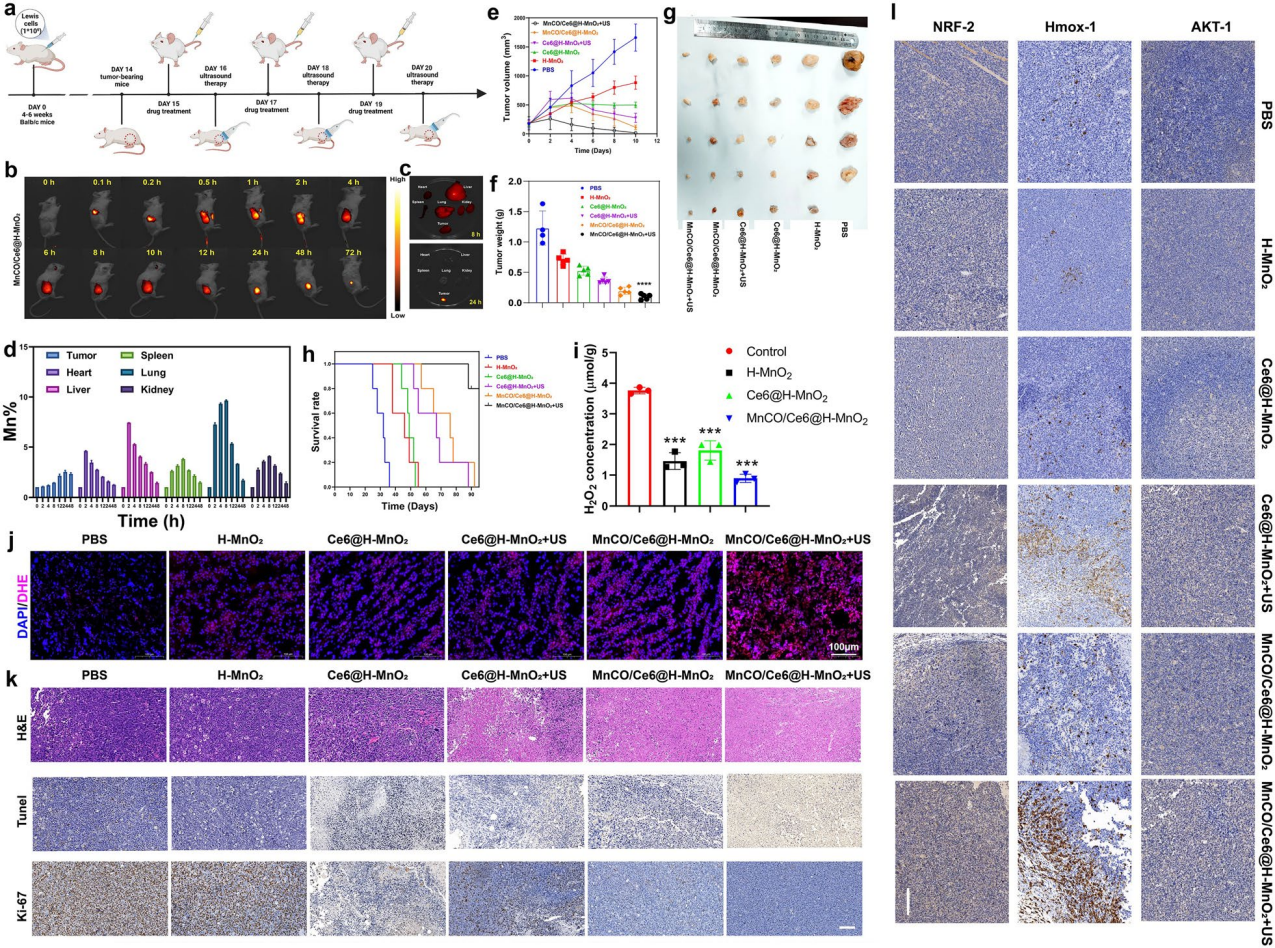
In vivo anticancer therapy evaluation on the combination of the maximumly-elevated ROS accumulation with long-lived CO release cascade catalysis-unlocked anti-tumor therapy. **a** Schematic representation of anticancer experimental procedures on the animal model. **b** In vivo time-correlated fluorescence images of Lewis tumor-bearing mice after intravenously administering Cy5.5-MnCO/Ce6@H-MnO_2_ (2 mg/kg equivalent Cy5.5). **c** Ex vivo fluorescence images of tumor, heart, liver, spleen, lung and kidney harvested from MnCO/Ce6@H-MnO_2_-treated mice at 8 h and 24 h, respectively. **d** Time-dependent Mn levels in tumor and normal organs that were determined by ICP-AES. Error bars indicate standard deviation (n = 3). **e** Time-correlated tumor volume variation profiles after different treatments. **f**, **g** Tumor weights (**f**) and digital photographs (**g**) after the end of the experimental period in varied treatment groups. **h** Surviving curves of Lewis tumors-bearing mice in various treatment groups (n = 5). **i** H_2_O_2_ levels in tumor that experienced different treatments. Error bars indicate standard deviation (n = 3). **j** CLSM images of DAPI&DHE co-stained tumor slices after different treatments for monitoring ROS level using ROS indicator (i.e., DHE), and scale bar: 100 μm. **k** Histological and immunohistochemical images of H&E, TUNEL, and Ki67 tests for the isolated tumor sections after different treatments, and Scale bar: 50 μm; **l** Histological and immunohistochemical analysis of AKT-1, Hmox-1, NRF-2 tests for the isolated tumor sections after different treatments, and Scale bar: 100 μm. H-MnO_2_ concentration: 5 mg/kg; ultrasound parameters: 1.0 MHz, 1.0 W/cm^2^, 15 s per time with 8 times for 120 s in total. Data presented as mean ± s.d. (n = 5). The mice in all experimental groups were euthanized and sampled on the 23rd day following injection of tumor cells. Moreover, symbol markers were employed to indicate instances of premature euthanasia resulting from rapid tumor growth, in accordance with principles of animal ethics.The statistical significance of differences was conducted with a Student’s t-test, and ****P < 0.0001

The in vitro cascade catalytic therapeutic consequences based on the engineered MnCO/Ce6@H-MnO_2_ nanocatalysis encourage the in vivo Lewis lung cancer recession. The detailed experiment protocol is drawn (Fig. 6a**),** and the common intravenous administering for lung cancer in clinics was used since it outperformed atomization absorption in evading exhalation-arised material loss and pulmonary mucosa and dense blood barriers-inhibited delivery. Firstly, in vivo biodistribution of nanoplatforms was monitored through labeling MnCO/Ce6@H-MnO_2_ nanoparticles with Cy5.5 since high accumulation of nanoplatforms in tumor is the premise of acquiring excellent treatment outcomes. Results clearly find that although the accumulation level of MnCO/Ce6@H-MnO_2_ firstly increases and subsequently gradually decreases within 72 h after injection (Fig. 6b), the retention in tumor is still kept at much higher level after 24 h post-injection even though the nanoplatforms in other organs are eliminated (Fig. 6b, c). Correspondingly, semi-quantitative fluorescence image analysis shows that Cy5.5 fluorescence signal is strong after 2 h post-injection at the site of tumor in MnCO/Ce6@H-MnO_2_ treated mice, and reaches the largest signal at 12 h (Figure S13). We also determined the biodistribution of MnCO/Ce6@H-MnO_2_ in vivo at various time points post-intravenous injection using ICP-AES analysis. Experimental results reveal that the material accumulates rapidly in normal organs (heart, liver, spleen, lungs, and kidneys) within 2–4 h post-injection, but is subsequently metabolized. After 48 h post-injection, the Mn level in all normal organs returns to that at pre-injection and approaches baseline levels (Fig. 6d and S14). In contrast, there is a gradual increase in material accumulation at tumor sites over time, which peaks at around 24 h and remains relatively a high level until 48 h. These results further underscore the highly-effective delivery and accumulation properties of this material specifically in tumors.

Depending on the high accumulation, a significant shrinkage after treatment is found, and the MnCO/Ce6@H-MnO_2_ + US group receives the largest tumor inhibition rate (94.3%) (Figs. 6e and S15), which is much higher than MnCO/Ce6@H-MnO_2_ (77.2%) and Ce6@H-MnO_2_ + US (55.7%). After sacrifice at the end of experimental period, tumors were collected, photographed and weighed. Tumor weights and corresponding digital photographs of representative mice in each group further demonstrate that the MnCO/Ce6@H-MnO_2_ + US treatment significantly inhibits tumor progression, resulting in the smallest tumor weight and volume (Fig. 6f, g), with no significant variation in body weight observed (Figure S16). Further, the survival rate of treated mice was recorded, and the MnCO/Ce6@H-MnO_2_ + US group makes a huge step to prolong the survival time (Fig. 6h), suggesting that the constructed nanotherapeutic system exerts the extremely pivotal effects on lung cancer recurrence inhibition and prognosis improvement in mice. To determine whether the H_2_O_2_ concentration is adequate for H-MnO_2_-induced CDT, the H_2_O_2_ levels were traced. It is clearly found that the concentration of H_2_O_2_ in tumor is approximately 4 μmol/g, but after treatments with H-MnO_2_-contained nanoplatforms, the H_2_O_2_ level significantly declines (Fig. 6i), suggesting the H_2_O_2_ reaction with H-MnO_2_. This result also validates that the H_2_O_2_ level in Lewis lung cancer is sufficient for triggering CDT, which is consistent with previous reports [40, 41]. Subsequently, in vivo ROS inspection was implemented and results reveal that the depleted H_2_O_2_ is converted into ROS in H-MnO_2_ group. More significantly, the SDT/CDT process in MnCO/Ce6@H-MnO_2_ + US group stimulates the most ROS production, which thus is expected to favor more CO birth via the cascade catalysis process (Fig. 6j).

To figure out the therapeutic rationales, tumor sections were inspected after staining with hematoxylin and eosin (H&E), TUNEL, and Ki-67 histochemical staining after various treatments (Fig. 6k, S17). The most large-area apoptosis/necrosis regions in H&E-stained images, the considerably-enhanced apoptosis in TUNEL-stained images, and the most significantly-suppressed cell expansion in Ki-67 test are obtained in tumors in the MnCO/Ce6@H-MnO_2_ + US group (Fig. 6k). The image clearly demonstrates that the high expression of the brown area corresponds to a high expression of the associated protein. Furthermore, upon comparing the results of the experimental group with those of MnCO/Ce6@H-MnO_2_ group and Ce6@H-MnO_2_ + US group, it is evident that Ki67, a proliferation-related protein, exhibits weak expression in the experimental group while there is a more significant expression of corresponding apoptosis-related proteins, revealing that the sequential treatment can expedite the deaths of cancer cells and oppose the replication of cancer cells. Beyond it, we further stained the tumor sections with AKT-1, HMOX-1 and NRF-2 antibodies to examine their expression (Fig. 6l, S17). Results show that the protein expressions of AKT signaling pathways including AKT-1, HMOX-1 and NRF-2 indicators are remarkably up-regulated, which is consistent with in in vitro WB analysis. It is noteworthy that the upregulation of pathway-related proteins in the experimental group not only significantly differed from that in the control group, but also exhibited distinct differences compared to those in the MnCO/Ce6@H-MnO_2_ group and Ce6@H-MnO_2_ + US group.The inspiring results further verify the mechanism feasibility of such a maximumly-elevated ROS accumulation and long-lived CO release cascade catalysis-unlocked anti-tumor therapy, that is, inhibiting the mitochondrial pathway and activating AKT signaling pathway.

**Fig. 7:**
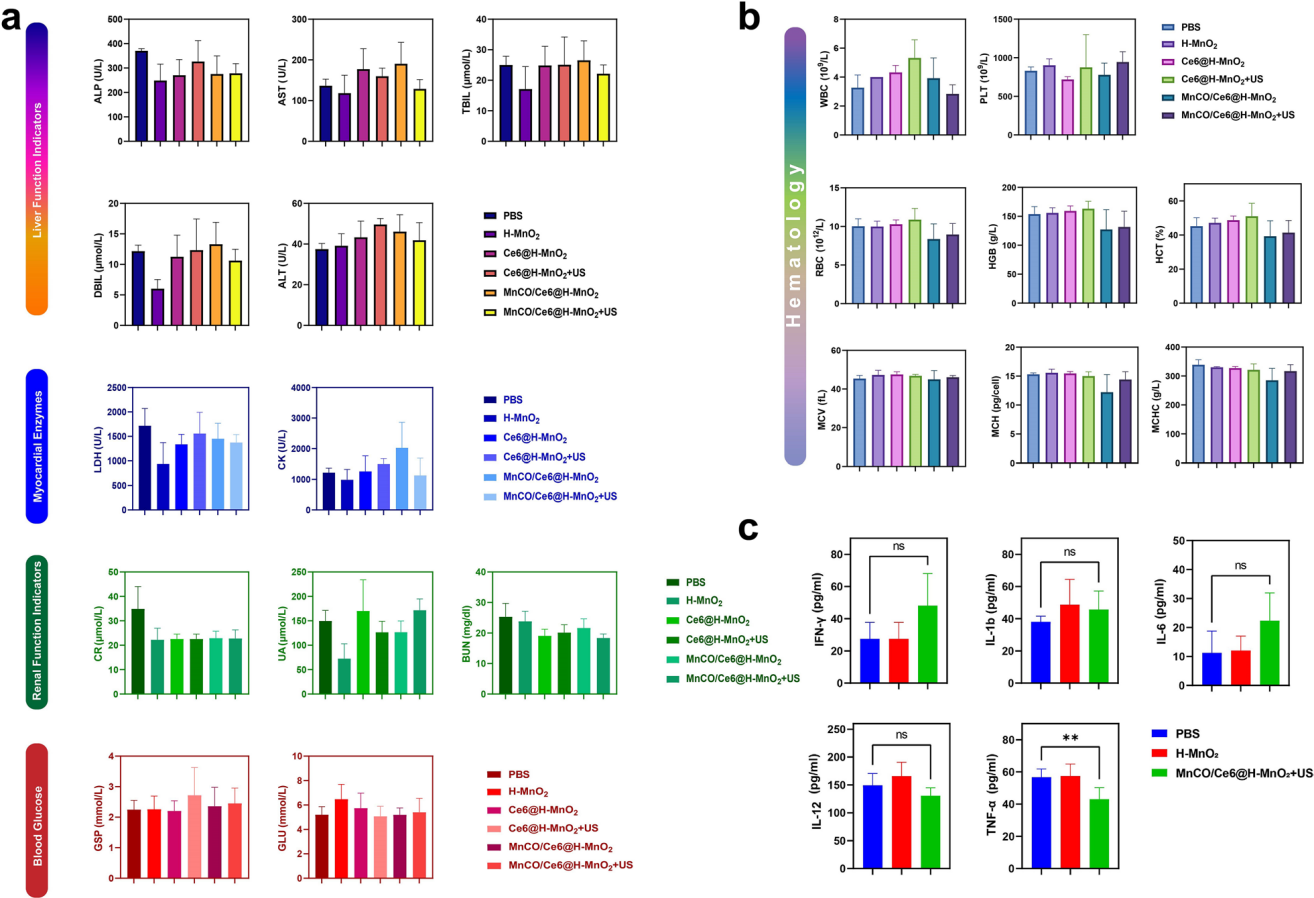
The criteria for evaluating biosafety in vivo. **a** Blood biochemistry analysis of balb/c mice that experiencing various treatment options. ALP: alkaline phosphatase; AST: aspartate aminotransferase; TBIL: total bilirubin; DBIL: direct bilirubin; ALT: alanine aminotransferase; LDH: lactic dehydrogenase; CK: creatine kinase; CR: blood creatinine; UA: uric acid; BUN: urea nitrogen; GSP: glycosylated serum protein; GLU: glucose. **b** Hematology data of balb/c mice that experiencing various treatment options. WBC: white bold cell; PLT: platelet; RBC: red blood cell; HGB: hemoglobin; HCT: hematocrit; MCV: mead corpuscular volume; MCH: mead corpuscular hemoglobin concentration; MCHC: mean corpuscular hemoglobin concentration. **c** Serum levels of some typical cytokines in balb/c mice treated with PBS, H-MnO_2_ as well as MnCO/Ce6@H-MnO_2_ + US nanoparticles

To evaluate the therapeutic safety, we performed H&E staining of heart, liver, spleen, lung and kidney of the injected mice, and results show that there is no evidence of significant organ damage in the MnCO/Ce6@H-MnO_2_ + US treatment group at the tested dose reflecting the good biocompatibility of MnCO/Ce6@H-MnO_2_ + US (Figure S18). Meanwhile, blood samples were collected from normal Bala/c mice after intravenous injection of PBS or MnCO/Ce6@H-MnO_2_ + US for hematological and blood biochemical tests. There is no prominent difference in serum indexes between the MnCO/Ce6@H-MnO_2_ + US group and the normal mice, further confirming that the safety of MnCO/Ce6@H-MnO_2_ (Figs. 7a and b). In addition, cytokines in serology were determined by enzyme-linked immunosorbent assay (ELISA) and MnCO/Ce6@H-MnO_2_ + US is disabled to induce prominent abnormalities in IL-1b, IL-12, IL-6 and IFN-γ levels. Although TNF-α is different between the two groups, the expression level remains within the normal range. These results also prove that MnCO/Ce6@H-MnO_2_ + US has no significant cytokine secretions (Fig. 7c). The accumulation of manganese in the body may lead to neurotoxicity. To further investigate the metabolism of carrier H-MnO_2_ in the body, we closely monitored the Mn content in the serum of mice in the treatment group (Figure S19). It was observed that during the administration period, there was a slight fluctuation in Mn content in mouse serum, which increased compared to before administration but still remained within normal range. Around the fourth day after completing treatment, almost complete metabolism of manganese in serum was observed. On day 7 after treatment, we closely monitored serum manganese levels and simultaneously euthanized mice to measure Mn content in their heart, liver, spleen, lungs, and kidneys. The results showed no statistically significant difference between manganese levels in these organs compared to those of normal mice(Figure S20).These biological effects initially prove that MnCO/Ce6@H-MnO_2_ + US has excellent biocompatibility, excellent biosafety and no obvious toxic side effects since their components have been validated to be safe [42–44], holding high potentials in clinical translation.

## Discussion

In recent years, significant progress has been made in the research of gas therapy for lung cancer, with mounting evidence supporting the anticancer effects of exogenous low-concentration carbon monoxide (CO). Recent reports have demonstrated that CO can enhance tumor sensitivity to chemotherapy and prevent doxorubicin-induced cardiotoxicity. Additionally, studies suggest that inhalation of exogenous CO may induce autophagy in lung cancer cells, leading to anti-tumor effects. However, there is limited research on utilizing nanomaterials for delivering the gas therapy in lung cancer treatment [45, 46].

Carbon Monoxide-Releasing Molecules (CORMs) have been developed as pharmaceutical agents to replace inhaled carbon monoxide therapy. CORMs are composed of central transition metals such as iron, manganese, or cobalt, coordinated with carbon monoxide as a ligand. This unique structure enables CORMs to effectively store and deliver controlled amounts of carbon monoxide to cells and biological environments. However, their limitations include short half-life, inadequate distribution control, and potential biological toxicity [47]. In recent years, the utilization of polymers and inorganic nanoparticles as efficient carriers for CORMs has the potential to develop innovative, stimulus-responsive drug delivery systems, thereby enhancing the clinical application of CO more effectively. However, despite the favorable therapeutic effects of CORMs, it is important to note that the in vivo toxicity of metal residues remaining after CO release is a significant concern, which may result in liver and kidney dysfunction, cellular damage, and disruption of electron transfer pathways within cellular components [48].

In summary, we developed such a maximumly-elevated ROS accumulation and long-lived CO release cascade catalysis strategy and engineered an endogenous and exogeneous stimuli-triggered nanotherapeutic system for unlocking ROS-based anti-tumor therapy against lung cancer. The H-MnO_2_ carriers were validated to respond to endogenous acidic and H_2_O_2_-enriched milieu and pose structural collapse to release Ce6 and Mn_2_(CO)_10_, re-programme tumor microenvironment and trigger CDT to produce •OH, which synergized with exogenous ultrasound-triggered and Ce6-mediated sonocatalytic ^1^O_2_ birth to enable the maximum accumulation of ROS, addressing the 1st concern that current ROS-based anti-tumor therapy encountered. More significantly, cascade catalytic reaction between Mn_2_(CO)_10_ and ROS was accessible and leveraged to trigger CO release, which successfully switched short-lived ROS to long-lived CO and addressed the 2nd concern that ROS-based anti-tumor therapy faced. The long-lived CO release not only assisted to boost the anti-tumor efficiency of CDT and SDT, but also disarmed the tumor resistance to ROS therapy and improved the sensitivity, consequently enabling a considerably-elevated lung cancer recession and the prolonged survival rate mattering tumor recurrence and metastasis. Especially, the therapeutic mechanisms were resolved, where inhibiting mitochondrial pathway and activating AKT signaling pathway including AKT-1, HMOX-1 and NRF-2 were found to be responsible for the excellent anti-tumor consequences. Thus, such multichannel actions-unlocked cascade catalysis strategy for ROS accumulation and CO release paved a new pathway to treat lung cancer. Certainly, our research does possess certain limitations. Currently, all of our experiments have been exclusively conducted on animal models, and the translation of these findings into practical applications for the benefit of patients remains an area that requires further exploration. Moreover, although we have developed a novel and effective nanomaterial for lung cancer treatment, we have yet to compare its efficacy, cost-effectiveness, and other aspects with existing treatment options available for lung cancer. This necessitates additional research and investigation.

## Conclusions

In this report, an endogenous and exogeneous stimuli-triggered cascade catalysis strategy has been established to maximumly elevate ROS accumulation and evoke long-lived CO release to unlock ROS-based anti-tumor therapy against lung cancer after engineering a nanotherapeutic system wherein hollow manganese dioxide (H-MnO_2_) carriers to load chlorin e6 (Ce6) sonosensitizer and CO donor (e.g., Mn_2_(CO)_10_). Thus, this endogenous and exogeneous stimuli-triggered cascade catalysis strategy can serve as a general method to enrich ROS and trigger CO release against refractory cancers, which will enlighten more interests among clinicians and researchers in clinical and fundamental research.

All materials, experimental methods and figures S1-S13 are included in supporting information.

### Supplementary Information

Additional file 1.

## Data Availability

All data can be obtained from corresponding authors upon reasonable request.
