# Supplementary material for: Endogenous and exogeneous stimuli-triggered reactive oxygen species evoke long-lived carbon monoxide to fight against lung cancer
Source: J Nanobiotechnology. 2024 Jul 16;22:416. doi: 10.1186/s12951-024-02688-x (PMC11253342; doi:10.1186/s12951-024-02688-x)
Supplement: Supplementary file 1 — Additional file 1. [file 12951_2024_2688_MOESM1_ESM.docx]

*Supplementary Materials for*

**Endogenous and Exogeneous Stimuli-Triggered Cascade Catalysis Evoke Long-Lived Carbon Monoxide to Fight against Lung Cancer**

**Supplementary figures**


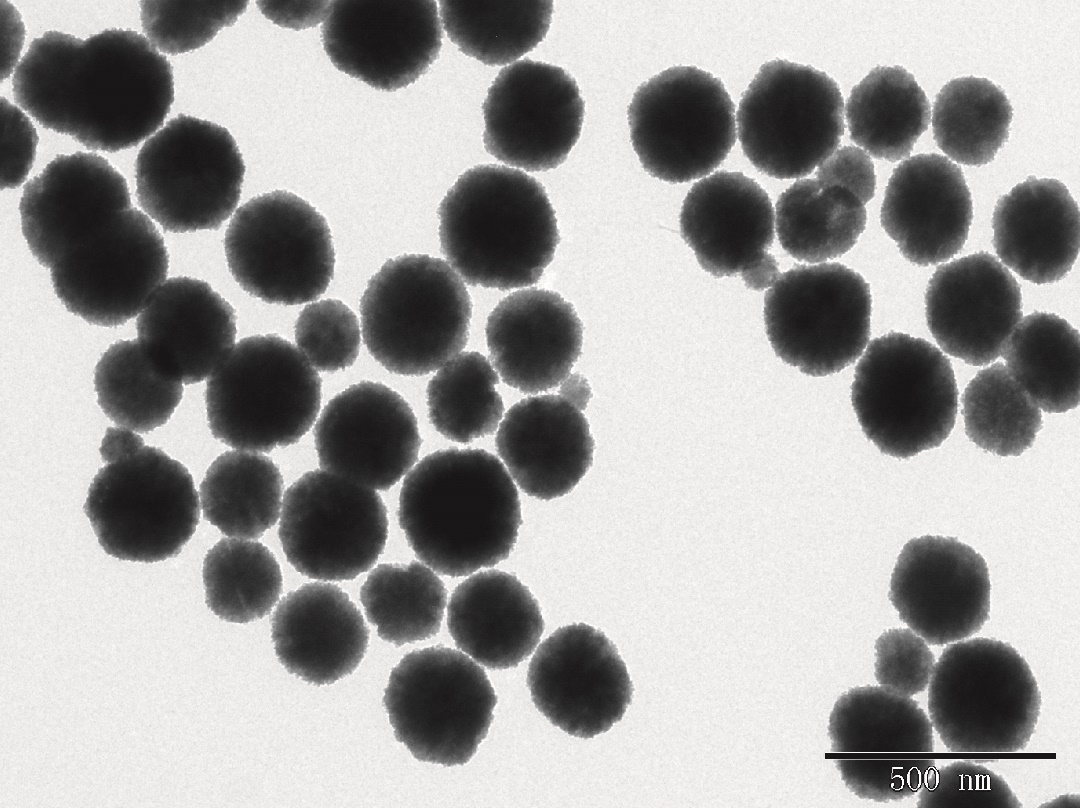


**Figure S1.** TEM image of monodispersed silica nanoparticles.


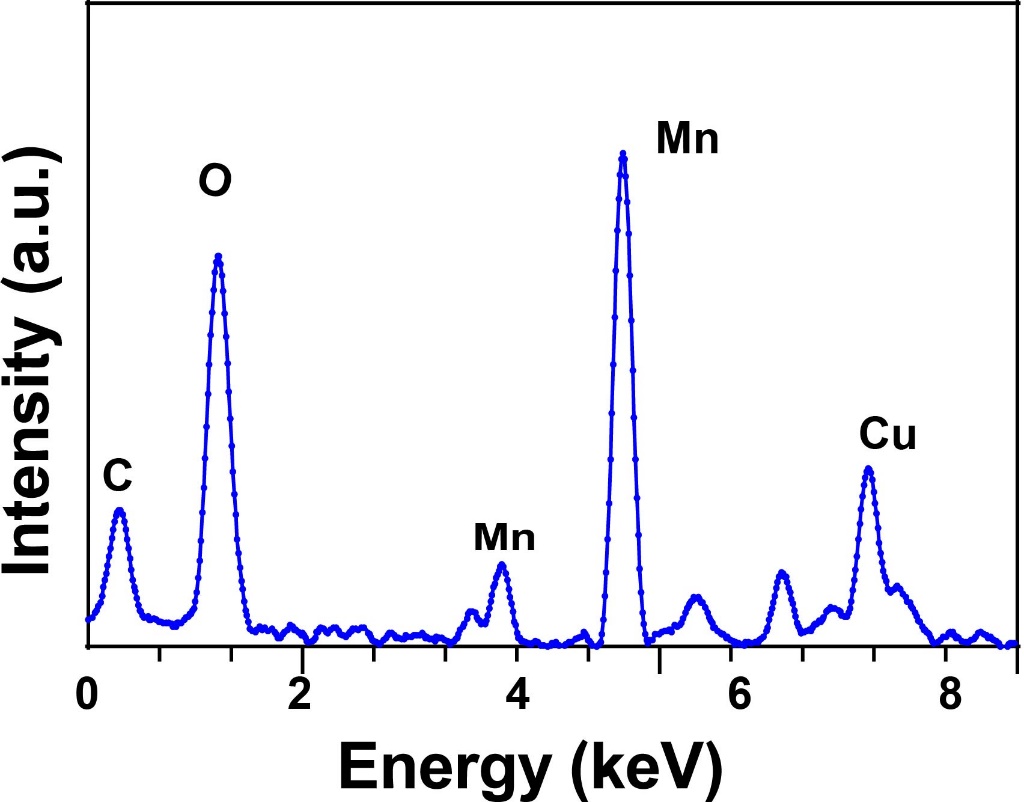


**Figure S2.** EDS analysis of HMnO_2_ NPs

**Figure S3.** Mn_2_(CO)_10_ loading capacities on H-MnO_2_ (w/W%) with increasing Mn_2_(CO)_10_ feeding concentrations. The loading capacity of MnCO instantly increased with a saturation level of 40.5% (Mn_2_(CO)_10_: H-MnO_2_, w/W%) in line with the feeding elevation of Mn_2_(CO)_10_ concentrations.

**Figure S4.** Ce6 loading capacities on H-MnO_2_ (w/W%) with increasing Ce6 feeding concentrations. The loading capacity of Ce6 instantly increased with a saturation level of 32.6% (MnCO:H-MnO_2_, w/W%) in line with the feeding elevation of Ce6 concentrations.

******

**Figure S5.** The drug loading rate of the nanomaterials was observed after different soaking durations. It is evident that the materials achieved saturation in drug loading within approximately 24 hours, thus this loading condition was selected for subsequent experiments.


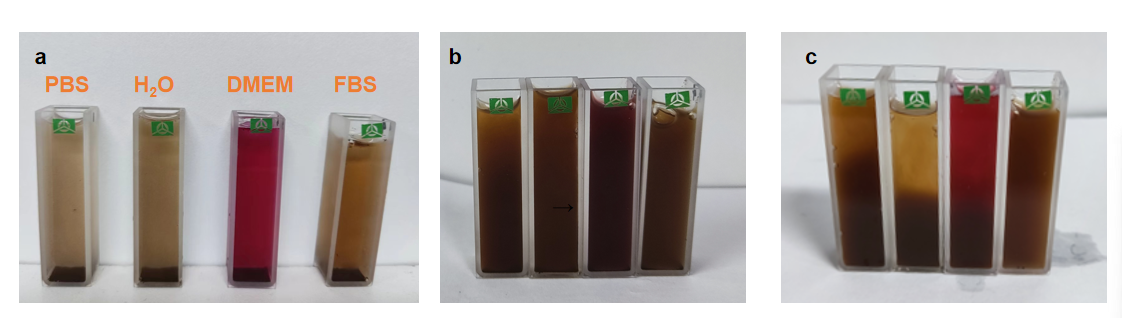


**Figure S6.** a.Materials of equivalent quality in various solutions (in a state of 0 hours without thorough mixing). b.The post-mixing state of the material in various solutions. c.The material state following 24 hours of blending different solutions.


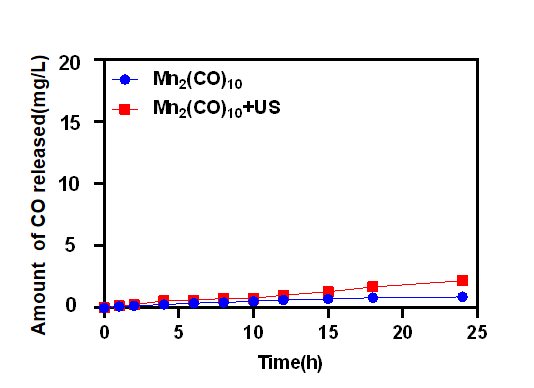


**Figure S7.** In vitro accumulative burst amount of CO from Mn_2_(CO)_10_ at different conditions. US parameters: 1.0 MHz, 1.0 W/cm^2^, 15 s per time with 2 times for 30 s in total.Date are presented as means ± s.d. (n=3).


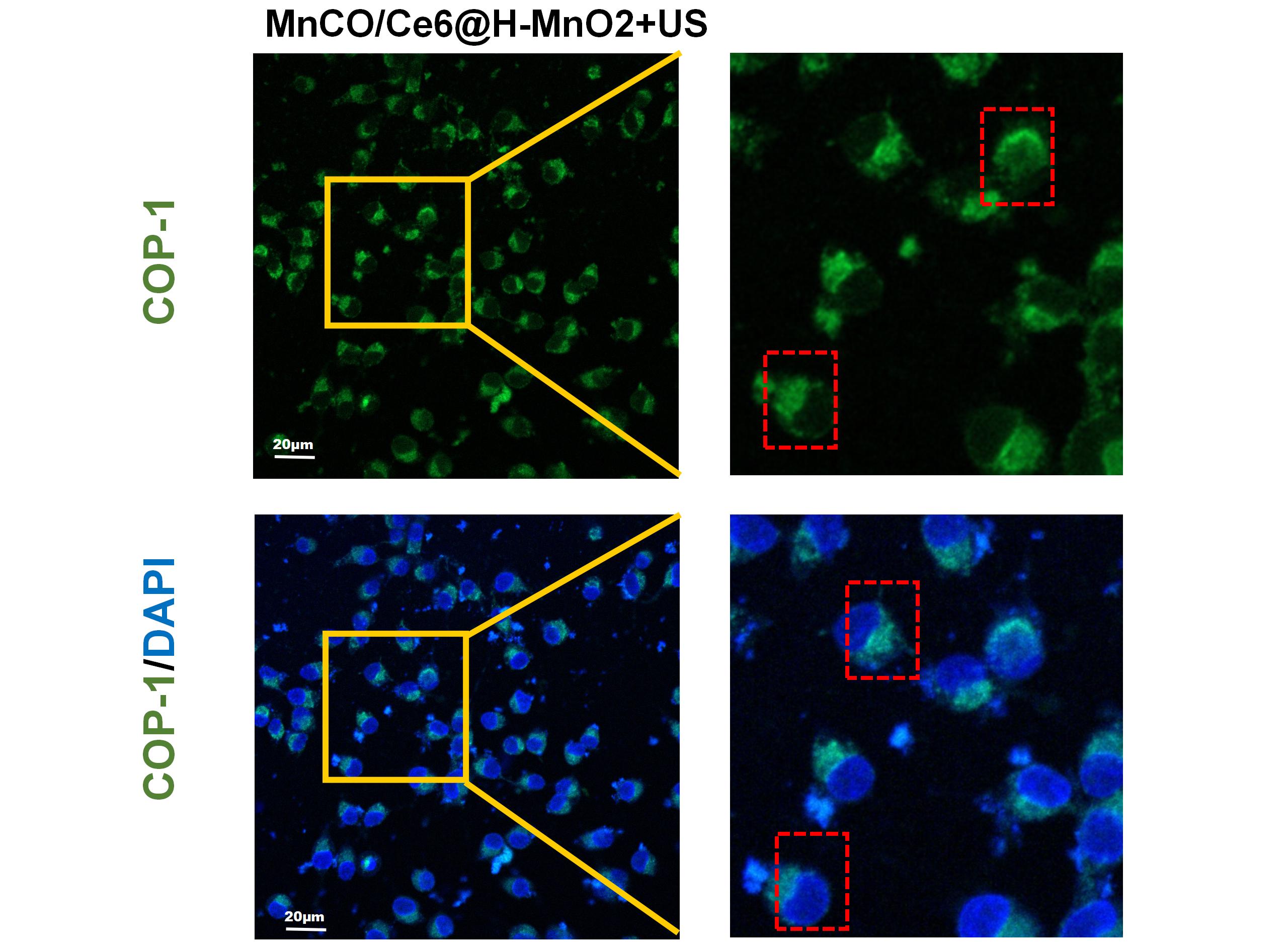


**Figure S8.**The local enlargement images of the Lewis cells in the experimental group were utilized for monitoring cellular CO levels using CO-1 as the fluorescent probe for CO detection.


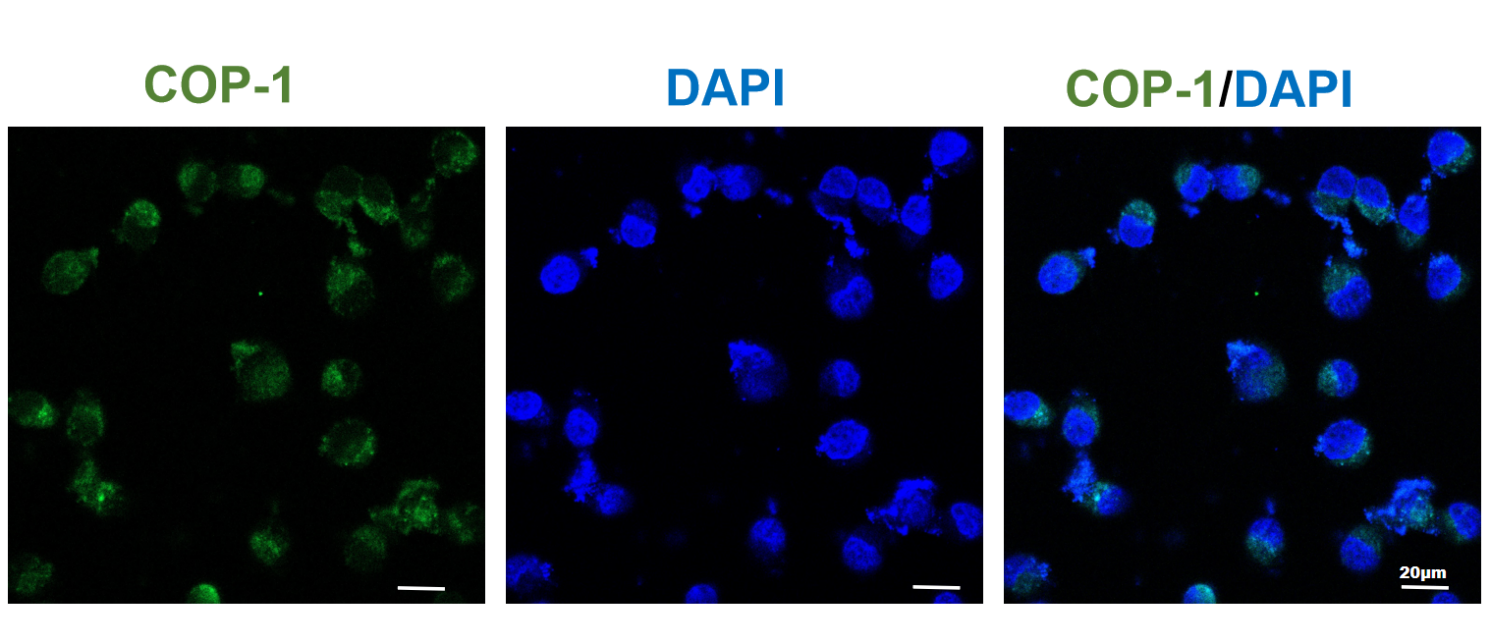


**Figure S9.**The probe localization map derived from confocal laser scanning microscopy (CLSM) images of Lewis cells was utilized for monitoring intracellular CO levels, with CO-1 serving as the fluorescent probe.


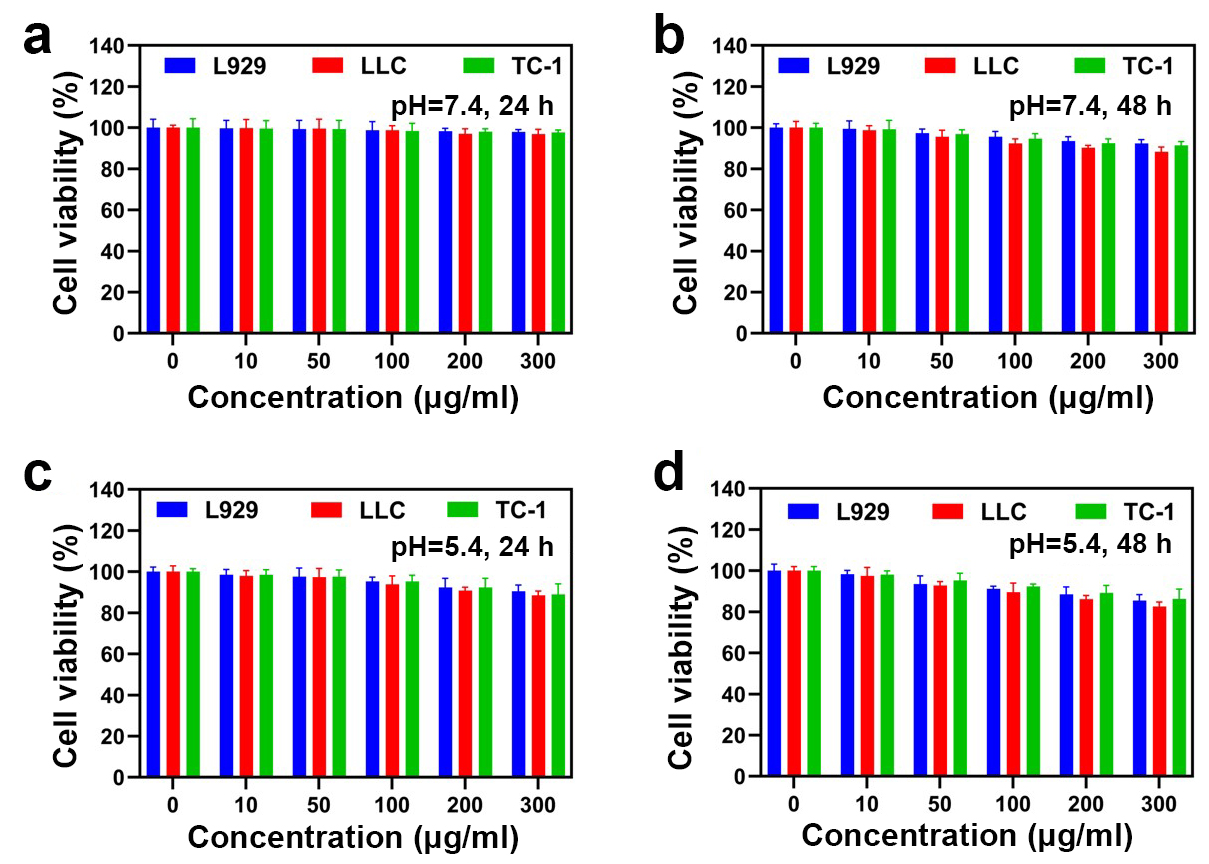


**Figure S10.** Relative viabilities of L929, Lewis (LLC) cells, TC-1 cells after incubation with H-MnO_2_ nanoparticles (concentration: 10, 50, 100, 200 and 300 μg/mL) at pH=7.4 or 5.4 for 24 h and 48 h, respectively.

**Figure S11.** The Lewis cells stained with calcium-chlorophyllin-AM and PI were subjected to various cohorts underwent a 24-hour treatment period, followed by quantitative analysis. Note, H-MnO_2_: 100 μg/mL; US parameters: 1.0 MHz, 1.0 W/cm^2^, 15 s per time with 4 times for 60 s in total.


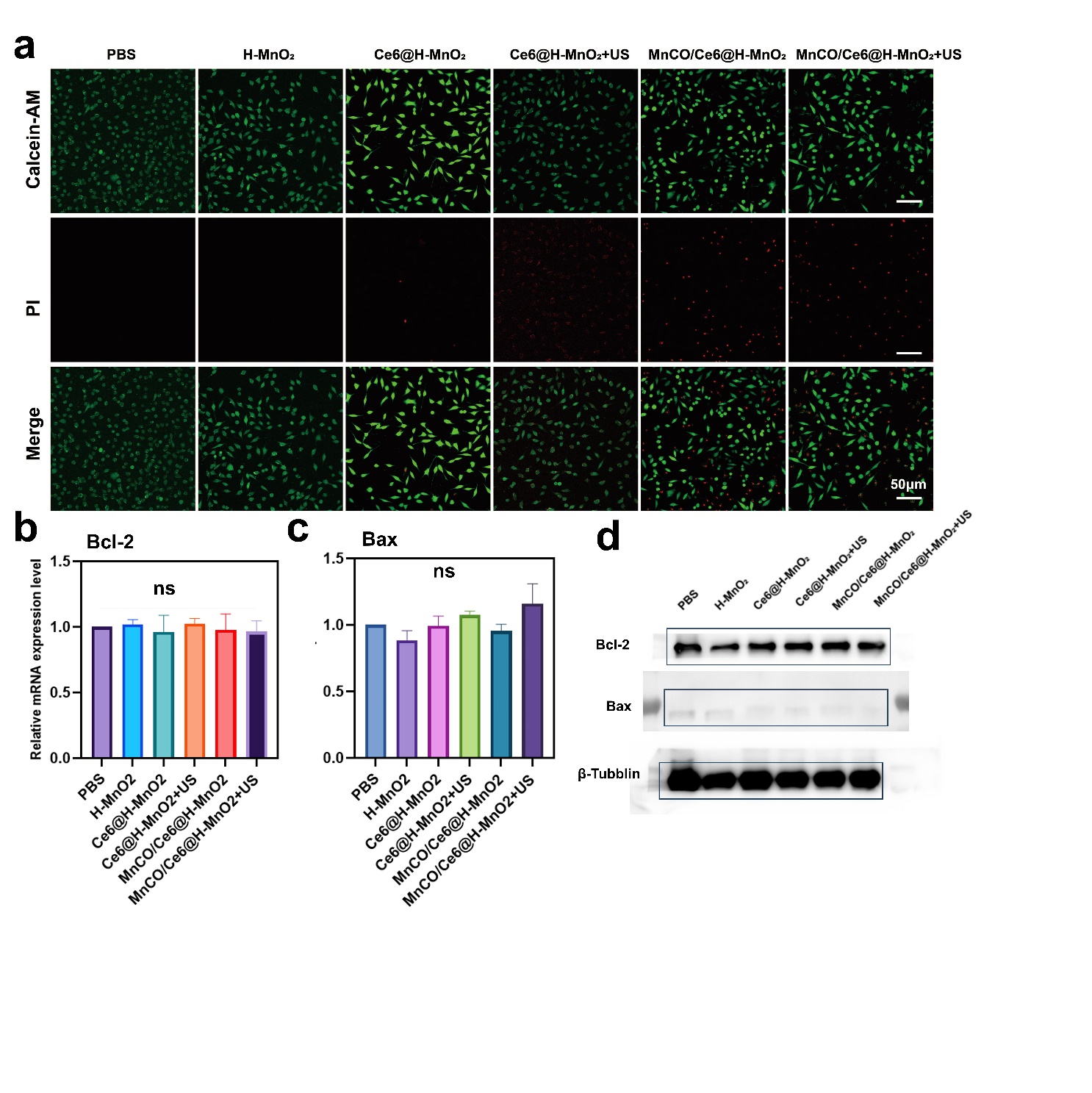


**Figure S12** (a)CLSM photos of calcein-AM/PI co-stained L929 cells that underwent various corresponding treatments.(b,c) Relative mRNA transcription levels of Bcl-2 and Bax in L929 cells that underwent varied treatments. (d) Western blot bands of Bcl-2 and Bax expression in L929 cells that underwent varied treatments.


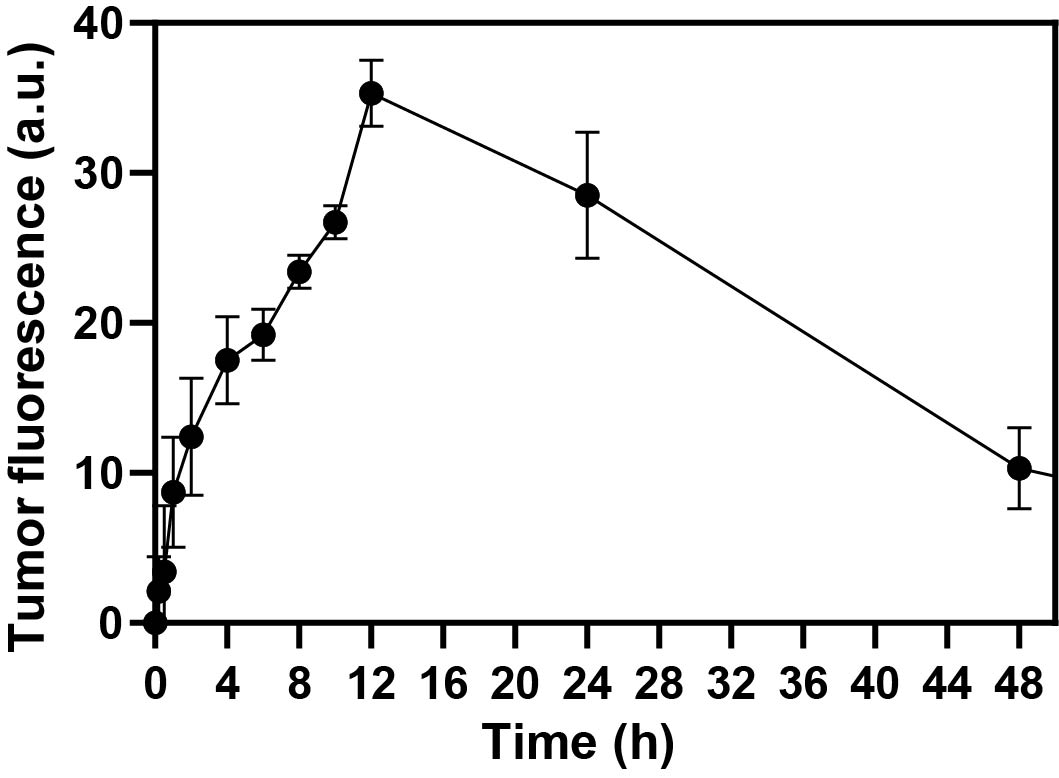


**Figure S13.** The relatively quantitative signal intensity at tumors as a function of various time intervals.


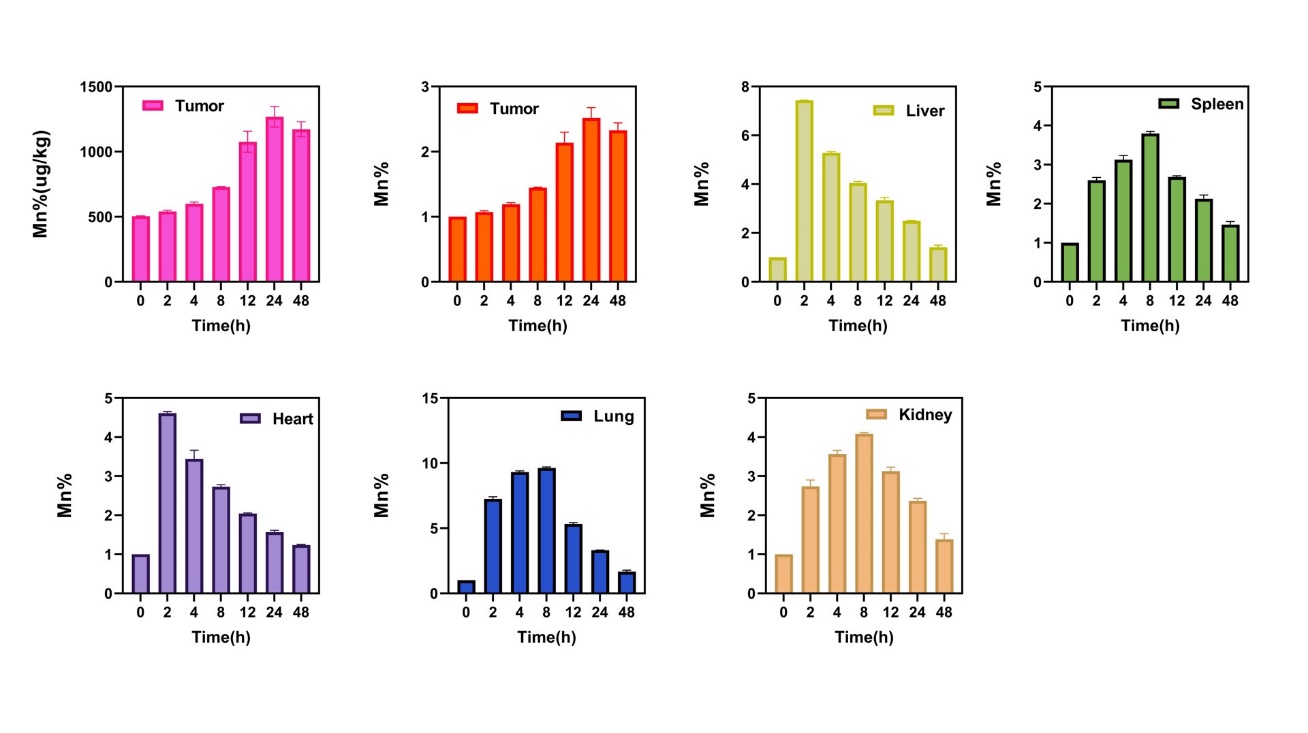


**Figure S14**.Time-dependent Mn levels in tumor and normal organs that were determined by ICP-AES. Error bars indicate standard deviation (n=3).


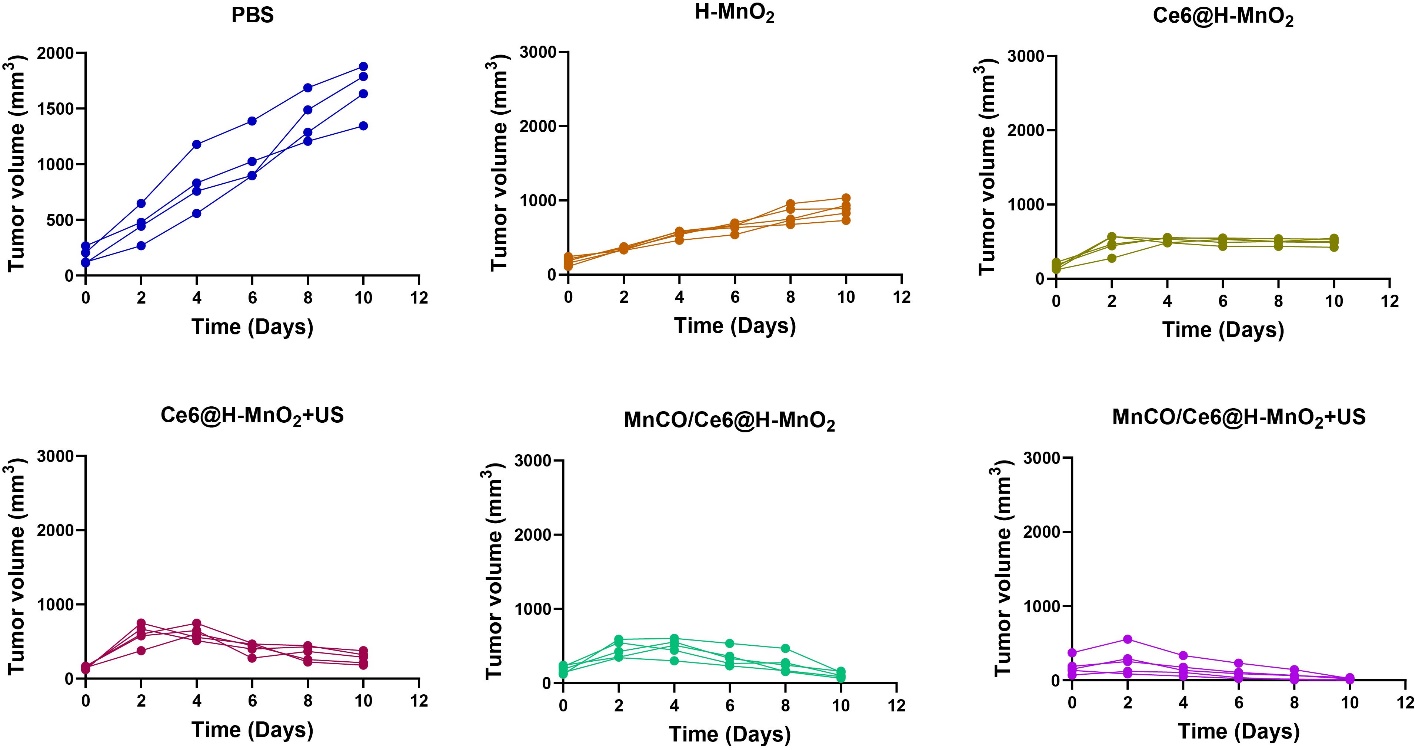


**Figure S15.** Lewis variation profiles of tumor volumes after intravenously administering the various formulations and undergoing subsequent ultrasonic therapy in Balb/c mice in different groups.


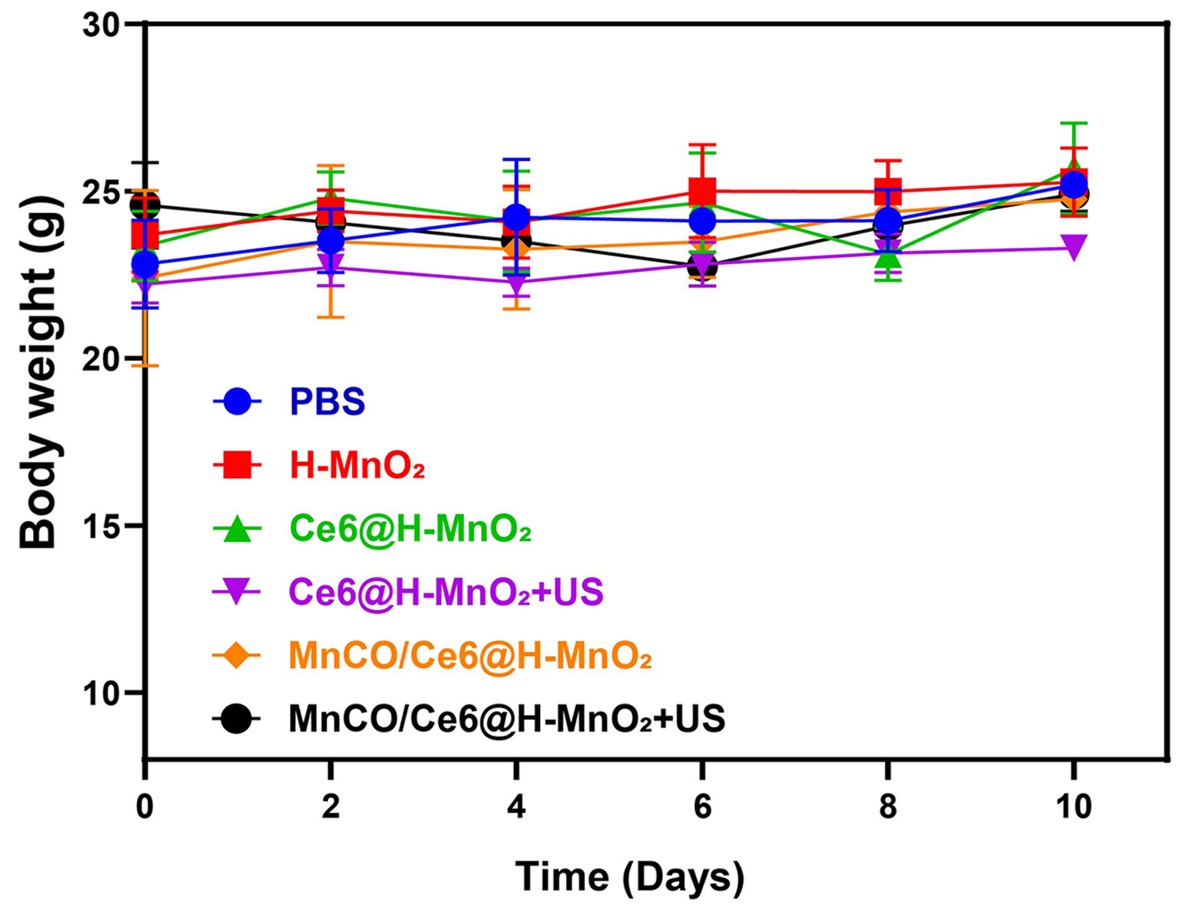


**Figure S16.** Time-correlated body weights of LLC tumor-implanted mice treated with various operations.


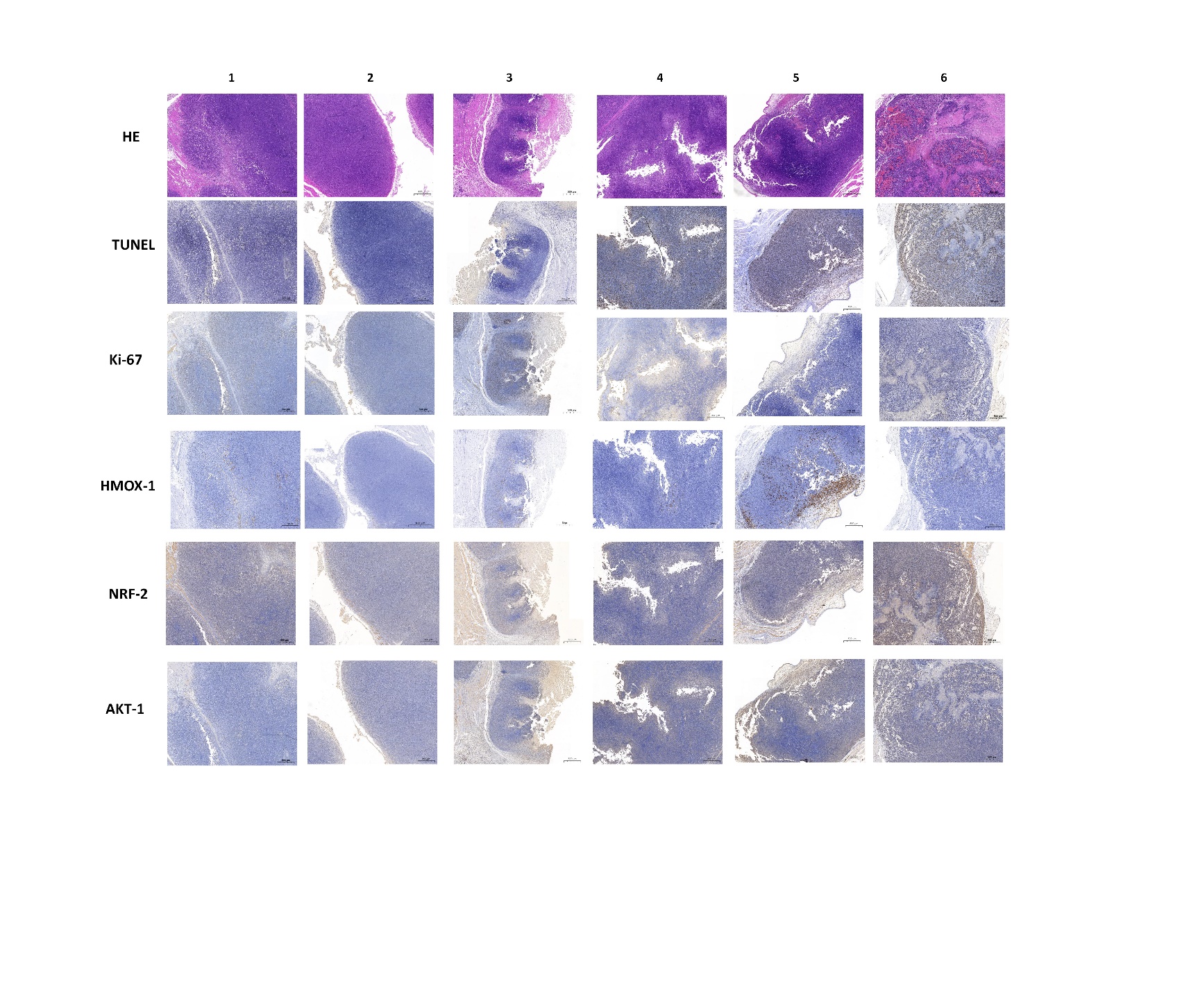


**Figure S17.**The full IHE image.1: PBS; 2: H-MnO_2_; 3: Ce6@ H-MnO_2_;4: Ce6@ H-MnO_2_+US; 5: MnCO/Ce6@ H-MnO_2_;6: MnCO/Ce6@ H-MnO_2_+US


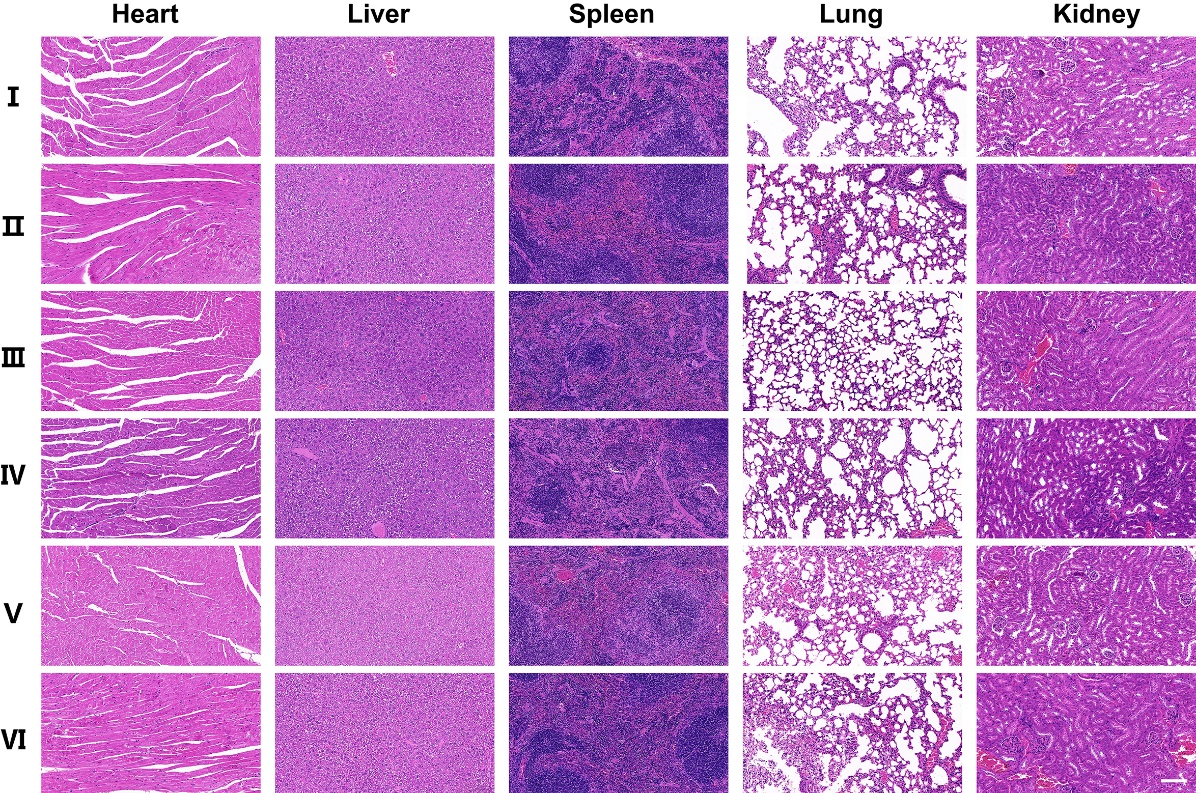


**Figure S18.** Pathological photos of H&E-painted main organs slices (heart, liver, spleen, lung and kidney) in the following groups. Scale bar: 50 μm. Ⅰ: PBS; Ⅱ: H-MnO_2_; Ⅲ: Ce6@ H-MnO_2_; Ⅳ: Ce6@ H-MnO_2_+US; Ⅴ: MnCO/Ce6@ H-MnO_2_; Ⅵ: MnCO/Ce6@ H-MnO_2_+US

**Figure S19**.Alterations in serum manganese levels following a 14-day treatment regimen in mice.Error bars indicate standard deviation (n=3).

**Figure S20**.The manganese content in the heart, liver, spleen, lungs, and kidneys was compared between the treatment group mice after completing all treatments for 7 days and normal 8-week-old mice.Error bars indicate standard deviation (n=3).
